# Modular 4WD agricultural robot for cutting, collection, and precision seeding: design and simulation-based evaluation

**DOI:** 10.1038/s41598-026-44388-6

**Published:** 2026-03-17

**Authors:** Abhishek Kumar, Shenoy Adithya Kamalaksha, R. Srividya, Mohammad Zuber, Kamarul Arifin Ahmad, Spoorthi Singh, Vishnu G. Nair

**Affiliations:** 1 Manipal Institute of Technology, Manipal Academy of Higher Education, Manipal, India; 2 Department of Aerospace, Faculty of Engineering, University Putra Malaysia, Serdang, 43300 Selangor Malaysia

**Keywords:** Autonomous Agricultural Robot, Modular Attachments, Grass Cutting, Debris Crushing, Precision Seeding, Differential-Drive, Coverage‐Path Planning, Boustrophedon, Monte Carlo Robustness Simulations, Slope‐Climb Analysis, Stone‐Ingestion Modelling, Battery‐Discharge Estimation, Finite‐Element Analysis (FEA), Engineering, Mathematics and computing

## Abstract

This paper presents a four-wheel differential-drive (4WD) autonomous platform that consolidates grass cutting, collection, leaf crushing, and precision seeding through modular, quick-release toolheads. A vertically stacked two-unit architecture separates the drive/blower subsystem in a steel-framed base from a high-capacity collection chamber; transparent panels aid inspection and service. System specifications are formalized, and operating energy budgets are modelled to predict runtimes across cutting (≈ 1.2 h), crushing (≈ 2.0 h), and seeding (≈ 8.0 h) modes. Coverage-path algorithms (zigzag, spiral, concentric) are simulated, with results confirming that the boustrophedon pattern achieves complete rectangular coverage with minimal redundancy. Robustness simulations quantify debris deflection (> 95% rejection), slope climb limits (≈ 25° at < 20% slip), and stone-ingestion probability (≈ 10%), validating operational resilience. Finite-element analysis of the steel and aluminum chassis demonstrates high safety factors (> 15) with negligible stress or deformation under representative static loads. Beyond robotic functions, composting pathways for collected biomass are outlined to close the loop on sustainability. While dynamic load events and hardware validation are deferred to future work, the results indicate that the proposed modular 4WD platform integrates cutting, collection, and seed delivery with serviceability, structural robustness, and environmental benefit, making it a promising candidate for campus and small-scale agricultural automation.

## Introduction

Agriculture, a cornerstone of human civilization, is currently undergoing a transformative phase driven by the integration of advanced technologies. The pressing challenges of labour shortages, the need for sustainable practices, and the demand for increased productivity have catalysed the adoption of automation and robotics in farming operations. Traditional farming methods, while effective in the past, often fall short in addressing the complexities of modern agriculture, such as precision, efficiency, and adaptability to varying field conditions. In this context, agricultural robotics has emerged as a promising solution to enhance productivity and sustainability. Robots equipped with advanced sensors and actuators can perform tasks ranging from soil preparation and seeding to harvesting and monitoring crop health. However, the diversity of agricultural tasks and the variability of field conditions necessitate robots that are not only capable but also adaptable. This has led to the exploration of modular designs in agricultural robotics, allowing for the customization and reconfiguration of robotic systems to suit specific tasks and environments. The integration of robotics into agriculture has been extensively studied, with a focus on developing systems that can perform specific tasks such as planting, weeding, and harvesting. However, the concept of modularity in agricultural robots has gained attention only in recent years. The authors, Xu et al.^1^ introduced the Modular Agricultural Robotic System (MARS), which emphasizes a modular architecture comprising interchangeable hardware and software components. This design allows for the customization of the robot to perform various tasks, demonstrating the potential of modularity in enhancing the versatility of agricultural robots.

Similarly, Guri et al.^2^ developed ‘Hefty,’ a modular reconfigurable robot designed to adapt to different agricultural tasks through the integration of various modules. Their work highlights the importance of modularity in facilitating the transition from research prototypes to practical field applications. In the realm of practical applications, Swarm Farm Robotics has implemented modular designs in their autonomous farm robots, enabling tasks such as fertilizing, weeding, and soil tilling. Their approach underscores the commercial viability of modular agricultural robots and their potential to revolutionize farming practices. The integration of robotics and Internet of Things (IoT) technologies in agriculture has led to significant advancements in precision farming, particularly in the domain of pesticide application. Traditional pesticide spraying methods often result in excessive chemical usage, environmental contamination, and health hazards to farm workers. To address these challenges, researchers have developed various IoT-enabled robotic systems aimed at optimizing pesticide application. Krishnaleela et al.^3^, introduced a multi-purpose agricultural pesticide spraying robot that leverages IoT for remote monitoring and control. The system utilizes sensors to detect environmental conditions and adjust spraying parameters, accordingly, enhancing efficiency and reducing chemical waste. Aagase et al.^4^ developed an IoT-enabled pesticide sprayer robot designed to replace manual labor in pesticide application. The robot incorporates ultrasonic sensors for obstacle detection and employs a microcontroller-based system for autonomous navigation and spraying. an autonomous agriculture robot for smart farming, capable of conducting semi-autonomous farm operations, including pesticide spraying, and provides information for tasks such as yield estimation and crop health monitoring^5,6^. Balasingham et al.^7^ presented SPARROW, a smart precision agriculture robot designed for weed control. The robot employs a vision-based autonomous navigation system and a spot spraying algorithm to detect and eliminate weeds efficiently. Although several multipurpose agricultural robots have been reported in literature, most exhibit significant limitations that hinder wide deployment. For instance, modular systems such as MARS^1^ emphasize flexibility but are bulky and primarily suited for research demonstrations rather than lightweight field use. Reconfigurable robots like Hefty^2^ focus on manipulation tasks in orchards and vineyards but do not integrate mowing or seeding functions. Weed-control platforms such as SPARROW^7^ achieve precise spraying but are restricted to task-specific operations, lacking true multifunctionality. Commercial systems like Farming GT^5^ offer high productivity but at the expense of very large mass and cost, making them inaccessible for small-scale farms. Furthermore, many prior studies rely only on static simulations or controlled trials, without validating robustness under diverse terrains, debris conditions, or extended endurance runs. These gaps underline the need for a lightweight, modular, and cost-effective platform that consolidates multiple tasks while maintaining operational resilience—an area directly addressed by the present work.

Furthermore, the design of multipurpose agricultural robots has been explored^8^, who proposed a robot capable of performing tilling, seeding, spraying, and watering. Their work demonstrates the feasibility of integrating multiple functionalities into a single robotic platform, although it lacks a modular approach. Despite these advancements, challenges remain in developing modular agricultural robots that are cost-effective, user-friendly, and capable of operating in diverse field conditions. This research aims to address these challenges by designing a modular multipurpose agricultural robot that combines flexibility, efficiency, and ease of use. Many universities and corporate campuses still rely on manual grass cutting and collection. To modernize this process, we propose an automated grass-cutter with integrated collection capabilities, an ideal solution for today’s landscaping challenges. To clarify the novelty of the proposed design, Table 4 provides a comparative summary of recent multipurpose agricultural robots, highlighting their key functionalities, payload capacities, and operational focus.

Our earlier work explored a 6WD variant; here we standardize on a 4WD configuration to reduce mass, drivetrain complexity, and associated energy losses. This paper presents the design and simulation-based assessment of a modular, four-wheel differential-drive agricultural robot that performs grass cutting, debris crushing, and precision seeding. The platform targets gaps in existing machinery by offering a rapidly reconfigurable two-unit architecture that reduces equipment swaps and downtime while improving coverage efficiency for small-scale and urban agriculture. Building on our earlier framework, which combined FEA, CFD with Darcy–Weisbach checks, Webots mobility simulation, and parametric endurance modelling for a suction-enabled grass-cutter^9^, the present study focuses exclusively on a four-wheel configuration and a simulation-only evaluation. Where appropriate, suction-path loss coefficients from the validated model in^9^ are reused solely to bound blower power and endurance and are not re-validated here. We integrate analytical power models, quasi-static FEA, coverage-path planning, and Monte-Carlo robustness analyses (debris deflection, slope/slip, and guard-window ingestion) to quantify energy budget, structural safety margins, and operational resilience. Collectively, these elements provide a transparent, reproducible basis for design decisions and mission planning, laying the groundwork for sustainable, data-driven groundskeeping practices.

The novelty of this work lies in integrating grass cutting, debris crushing, and precision seeding into a single, lightweight four-wheel differential-drive (4WD) platform supported by modular quick-release toolheads. Unlike prior multipurpose agricultural robots that either emphasize modularity but remain bulky (e.g., MARS) or are restricted to single tasks (e.g., SPARROW for spraying, Hefty for manipulation), the proposed design consolidates multiple ground-maintenance functions within a compact, serviceable chassis. A vertically stacked two-unit architecture separates the drive/blower subsystem from the high-capacity collection chamber, improving accessibility, transparency, and ease of maintenance. The contribution is further distinguished by simulation-based analysis beyond basic kinematics: coverage-path planning (zigzag, spiral, concentric) to optimize field efficiency, Monte Carlo robustness analyses to quantify debris deflection, slope/slip behaviour, and stone-ingestion probability, and energy-budget modelling to predict runtimes across cutting, crushing, and seeding modes. Finite-element analysis confirms high structural safety factors (> 15) for both steel and aluminium frames, demonstrating substantial design margins for real-world deployment. Finally, the inclusion of biomass valorisation through composting establishes an environmental dimension rarely addressed in agricultural robotics, thereby linking automation with sustainability. Collectively, these elements distinguish the proposed system as a novel and practical approach toward modular agricultural automation for campuses and small-scale farms. The next section details the 4-wheel platform architecture and specifications, followed by structural analyses, endurance modelling, autonomy/coverage methods, and simulation results.

### Grass cutter bot design

**Fig. 1:**
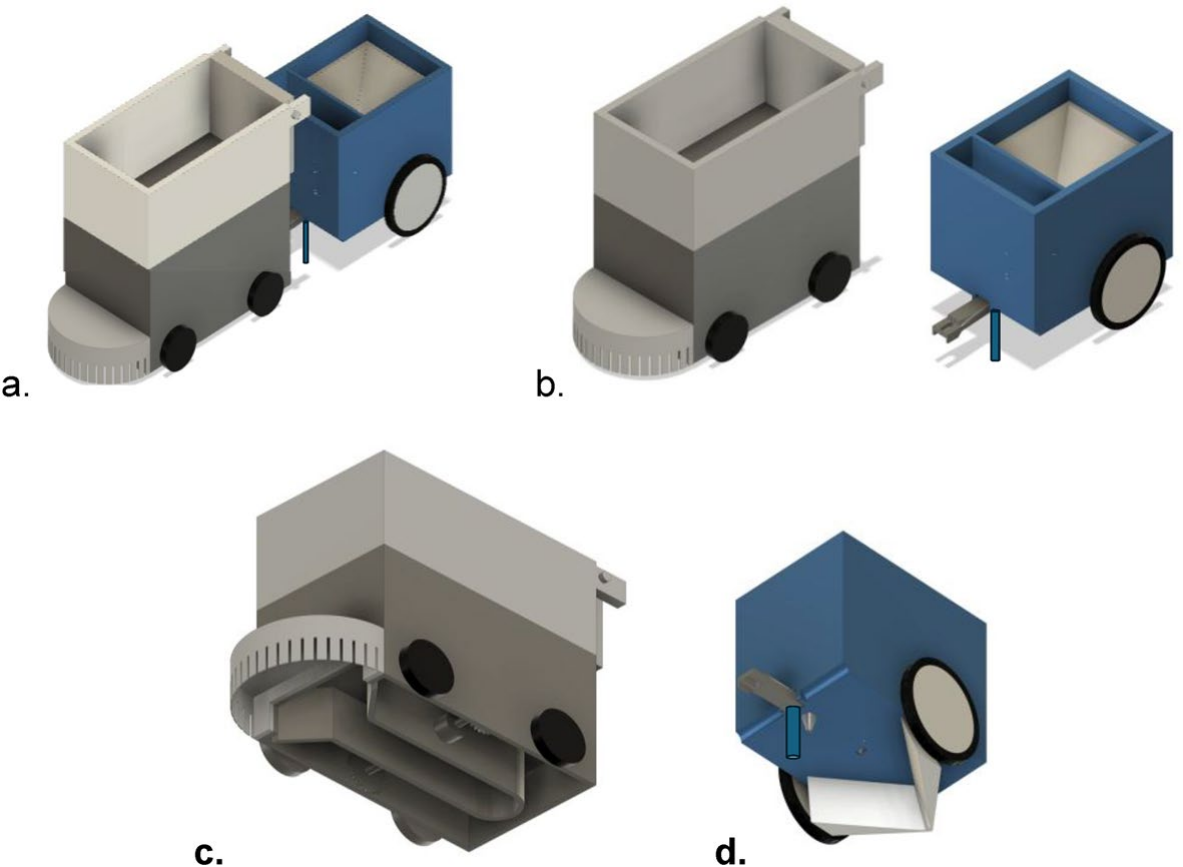
Isometric view of Autonomous : **a**. Grass Cutter bot with seed dispenser, **b**. Grass Cutter bot and seed dispenser shown separately. **c**. bottom view of grass cutter bot and **d**. bottom view of seed dispenser/Seeder Robot design with labelling of parts.

The platform is a four-wheel differential-drive (4WD) autonomous robot for cutting and collection on campus and estate grounds (see Fig. 1). A steel base frame with a ladder-type aluminium backbone provides bolted hard points for swappable modules cutter, debris guard, and an optional trailer hitch, All four wheels are powered (4 × 4); left and right wheel pairs are commanded independently (v_L_,v_R_) for straight tracking (v_L_=v_R_) and zero-radius pivots (v_L_ = − v_R_), maintaining skid-steer agility with a simple drivetrain. The design priorities are: (i) reliable operation on short turf and uneven paving; (ii) rapid module exchange without re-machining; (iii) manufacturability from standard sheet and structural sections; and (iv) safety by design via interlocks and guarded rotating elements. All analyses in this paper use this 4-wheel configuration. The chassis supports a vertically stacked, two-unit layout: a robust lower unit housing the drive, powertrain, and blower, and an upper high-capacity collection chamber with quick-release mounts for the trimmer, crusher, and seed-drill attachments. Key driver’s swath width, ground clearance, wheel configuration, and material selection are balanced with a semi-circular debris bumper, an adjustable cutter head, and the 4-wheel skid-steer kinematics to ensure reliable operation across varied terrains.

### Individual parts

- **Steel frame**: Primary load-bearing chassis with ladder-type aluminium backbone and bolted module mounts.
- **Transparent acrylic panels**: Protect internals while allowing visual inspection of electronics, drives, and airflow paths.
- **Electric motors**: Drive the wheels (propulsion) and the cutter head.
- **S-shaped blower**: Lifts clippings from the cutter zone and conveys them to the upper collection unit.
- **Adjustable-height trimmer**: Sets cut height for varying turf conditions.
- **Semi-circular bumper with slots**: Deflects stones/debris away from the cutter for safety and reliability.
- **Four motorised wheels (front + rear)**: Provide traction and steering via differential (skid-steer) control.

The platform integrates cutting, collection, crushing, and seeding within a single modular system suited to landscaping and light agricultural tasks. A vertically stacked, two-unit layout separates locomotion and powertrain (lower unit) from material handling (upper unit), while an optional trailer adds soil preparation and seeding capabilities.

- **Lower (Base) Unit**: A rugged steel chassis with acrylic cladding houses the drivetrain, power electronics, and an S-shaped centrifugal blower. During mowing, the blower’s bottom inlet entrains cut material through an internal duct and discharges it into the upper compartment. The **height-adjustable cutter** rides behind a slotted semi-circular bumper that shields the blades from stone impacts.
- **Upper (Collection) Unit**: A high-capacity collection box with a rear hatch enables rapid unloading, reducing downtime and improving field throughput.
- **Mobility & Drive (4 × 4 skid-steer)**: All four wheels are powered (front and rear pairs). Left-right side speeds (v_L_,v_R_) are commanded independently to deliver straight tracking (v_L_=v_R_) and zero-radius pivots (v_L_ = − v_R_​). This configuration combines towing capability for heavier attachments with reliable manoeuvrability on uneven ground.
- **Modular Toolheads**: Quick-release, front-mounted modules include a high-torque trimmer for live grass and a crusher for dry leaves and light debris. Standardised mechanical and power interfaces enable tool changes in minutes.
- **Seeding Trailer Attachment (optional)**: A two-axle trailer couples to the rear hitch. A hardened opener forms a seed furrow ahead of a rotating metering drum with calibrated holes that maintain dose uniformity. A trailing closure flap backfills the furrow for good seed–soil contact. A speed-matched drive keeps seed spacing consistent as forward velocity varies.
- **The trimmer height-adjustment system**, included in the CAD design, is intended to be driven by image-processing feedback, enabling adaptive cutting heights depending on grass length and environmental context (e.g., close-to-soil cutting in farmland vs. moderate-height trimming for lawns and campuses).

By consolidating cutting, collection, crushing, and seeding on one chassis, the system reduces equipment hand-offs and labour, supports rapid role changes without major rework, and simplifies maintenance via transparent covers and accessible service points. The 4 × 4 differential drive ensures stable, coordinated motion across turf, compacted paths, gravel, and light agricultural terrain. Safety-by-design measures (interlocks, guarded rotating elements) are incorporated throughout.

**Fig. 2:**
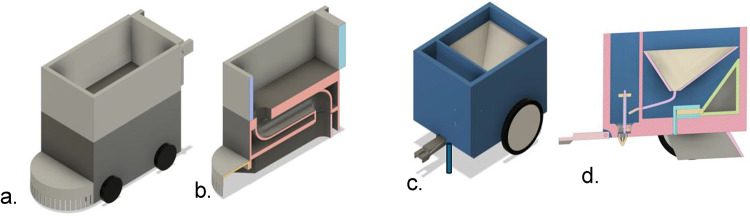
3D CAD models of the multipurpose agricultural robot modules.

(**a**) Grass-cutting autonomous robot with integrated cut-grass collector and S-shaped blower duct. (**b**) Cross-sectional view of the grass-cutting module showing internal ducting and collection chamber. (**c**) Seed dispenser unit for controlled seed distribution. (**d**) Cross-sectional view of seed dispenser showing hopper geometry and dispensing path.

Figure 2 illustrates the 3D CAD models of the major functional modules integrated into the multipurpose agricultural robot. The first module, shown in Fig. 2(a), is the grass-cutting autonomous robot equipped with an S-shaped blower duct. This duct geometry is designed to create a smooth airflow path that efficiently transports the cut grass from the mowing deck to the rear-mounted collection bin, minimizing blockages and preserving airflow velocity. The overall module incorporates a compact wheeled chassis for mobility and stability during field operations. Figure 2(b) presents a cross-sectional view of the grass-cutting module, revealing the internal configuration of the blower duct and collection chamber. The cross-section highlights how the duct curves facilitate uninterrupted grass flow while maintaining a compact internal layout. The second major module, shown in Fig. 2(c), is the seed dispenser unit, which ensures uniform seed distribution during sowing operations. The design includes a hopper for seed storage and an outlet mechanism for controlled dispensing. Figure 2(d) provides a cross-sectional view of the seed dispenser, detailing the hopper’s sloped geometry for continuous seed flow, the seed metering section for accurate dosing, and the outlet channel for precise placement in the soil. These modular designs allow for the integration of mowing, grass collection, and seeding functionalities into a single autonomous platform, enhancing versatility and operational efficiency in agricultural applications. Our previous chassis optimization (aluminium 6061-T6 backbone with acrylic panels) achieved ≈ 15% mass reduction while maintaining SF ≥ 2.0 under peak loads; we adopt that backbone here with module-specific reinforcements^9^.

### Seed dispenser (Modular attachment)

The seed dispenser is a compact, speed-matched metering unit that provides precise and uniform seed placement (Fig. 4). Bulk seed flows from the hopper into a rotating metering drum with calibrated holes. An adjustable gate/simulator trims the flow so ideally one seed is carried per hole; seeds then pass through a drop tube into the furrow formed by a hardened opener, and a trailing closure flap/press element restores soil contact. The module bolts to the robot’s rear hitch, draws power from the platform bus, and uses vehicle-speed feedback so the drum RPM tracks forward velocity, maintaining in-row spacing on varied terrain.

- **Metering principle**: Let v be forward speed (m·s^− 1^), s the target in-row spacing (m), n_h_ the number of holes per drum revolution, p the singulation probability (0–1), and G the motor : drum gear ratio. The required drum speed is.

For volumetric operation with cell volume V_c_ and bulk density ρ_b_:

- **Gate/simulator** : is an adjustable feature at the hopper–drum interface that ensures single-seed occupancy of each metering hole. The gate sets the bulk inflow by defining a narrow, height-controlled opening over the drum face, stabilizing the seed layer and preventing overfilling; the simulator, a bevelled lip, brush, or spring-loaded finger placed tangential to the drum path shears off “doubles” so only one seed remains in each cell before it reaches the drop tube. Its gap and angle are tuned to seed geometry (thickness, aspect ratio, surface texture) to minimize misses and doubles without cracking or scuffing seed. For durability and low friction, the contact edge is typically hardened metal or wear-resistant polymer with a small radius/chamfer. In operation, the gate/simulator complements the agitator by smoothing flow, desensitizing the metering rate to vibration and slope, and preserving in-row spacing accuracy across speed changes.
- **Calibration** : (1) Set desired s and measure v on turf. (2) Select n_h_​; start with *p* ≈ 0.95 via gate setting. (3) Compute RPM from the equation above. (4) Run over a tarp for a timed interval; count seeds to refine p and RPM. (5) Store a lookup (v→RPM) for closed-loop control.
- **Safety & serviceability.** Pinch points are guarded; a hopper-hatch interlock disables the drum when opened. Tool-free gate and drum swaps adapt the unit to different seed sizes.

**Fig. 3:**
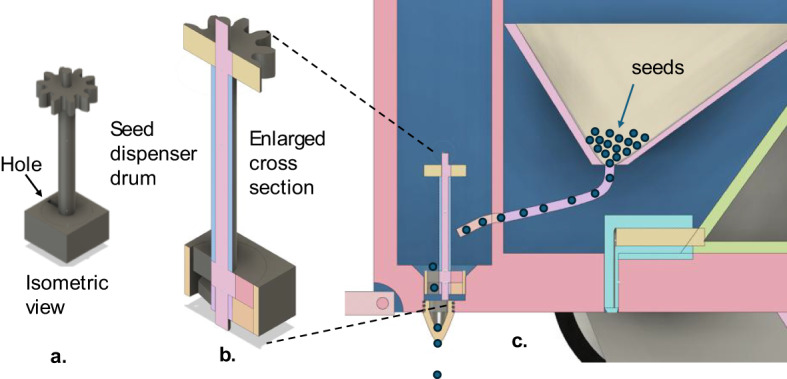
Seed dispenser module – **a**.sectioned isometric, **b**.enlarged cross-section, and **c**.chassis installation (seed flow shown).

Figure 3 shows detailed Sectioned-isometric of the dispenser body and star-hub shaft. Enlarged cross-section highlighting the hopper outlet, rotating metering drum with calibrated holes, adjustable gate/simulator, shaft and bearings, and the drop tube. Assembly section on the robot chassis showing hopper, chute, the furrow opener, and the trailing closure flap/press element. Blue markers trace the seed flow path from hopper → chute → drop tube → furrow. The dispenser operates in speed-matched mode so drum RPM tracks vehicle speed to maintain in-row spacing.

The metering performance of the seed dispenser is governed by the relationship between forward speed and drum rotation. For a desired in-row spacing s (m) at robot speed vvv (m·s − 1), the required seeds per second are N_seeds_=v/s. With a drum containing n_h_​ holes per revolution and singulation probability p, the necessary rotational speed is.

where G is the motor: drum gear ratio. For example, at v = 0.5vm/s, s = 0.05 m, n_h_=12, and *p* = 0.95, the dispenser requires N_seeds_=10 seeds/s and a drum speed of ≈ 52.6 RPM to maintain the spacing. Volumetric operation may be expressed as m˙=[ρ_b_*V_c_*(rev/s)], where V_c_​ is cell volume and ρ_b_​ is bulk seed density. Calibration is performed by running the unit over a fixed distance, counting seeds on a collection tarp, and adjusting gate and drum settings until the measured mean spacing sˉ, coefficient of variation (CV = σ_s_/sˉ), and miss/double rates fall within acceptable limits. This quantitative framework allows direct comparison of seed types, operating speeds, and gate settings, ensuring repeatable and accurate dispenser performance^16–18^.

### Adaptive trimmer height control with image processing

The schematic depicts the feedback loop between perception and actuation for adaptive grass cutting. A front-mounted RGB/stereo camera captures field imagery, which is processed by a grass-height estimation algorithm. The estimated height is compared against task-specific targets (e.g., full clearance for farmland, moderate cut height for lawns). Based on this decision logic, commands are sent to a vertical lift actuator that adjusts the trimmer head position in real time. This closed-loop system ensures context-aware cutting: reducing energy waste by avoiding unnecessary deep cuts, maintaining uniformity on landscaped lawns, and maximizing clearance in agricultural fields.

**Fig. 4:**
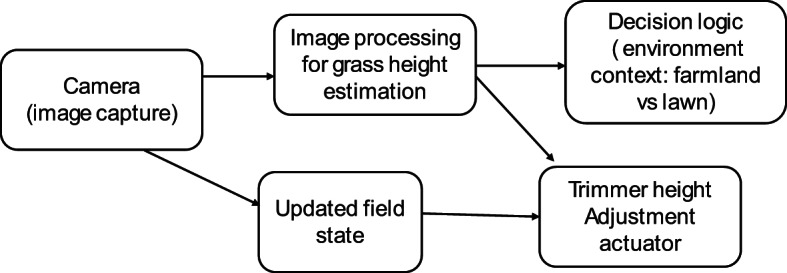
Conceptual schematic of adaptive trimmer height control.

Figure 4 illustrates the proposed adaptive trimmer height control framework. The CAD design includes a vertically adjustable trimmer head, and image-processing algorithms are being developed to estimate average grass height from field imagery. Depending on the environmental context, the decision logic sets the target cut height for example, close-to-soil trimming in farmland for maximum clearance, or moderate-height cutting in lawns to preserve turf quality. The actuator then adjusts the trimmer head in real time, achieving context-aware, energy-efficient, and uniform mowing^6^.

**Fig. 5:**
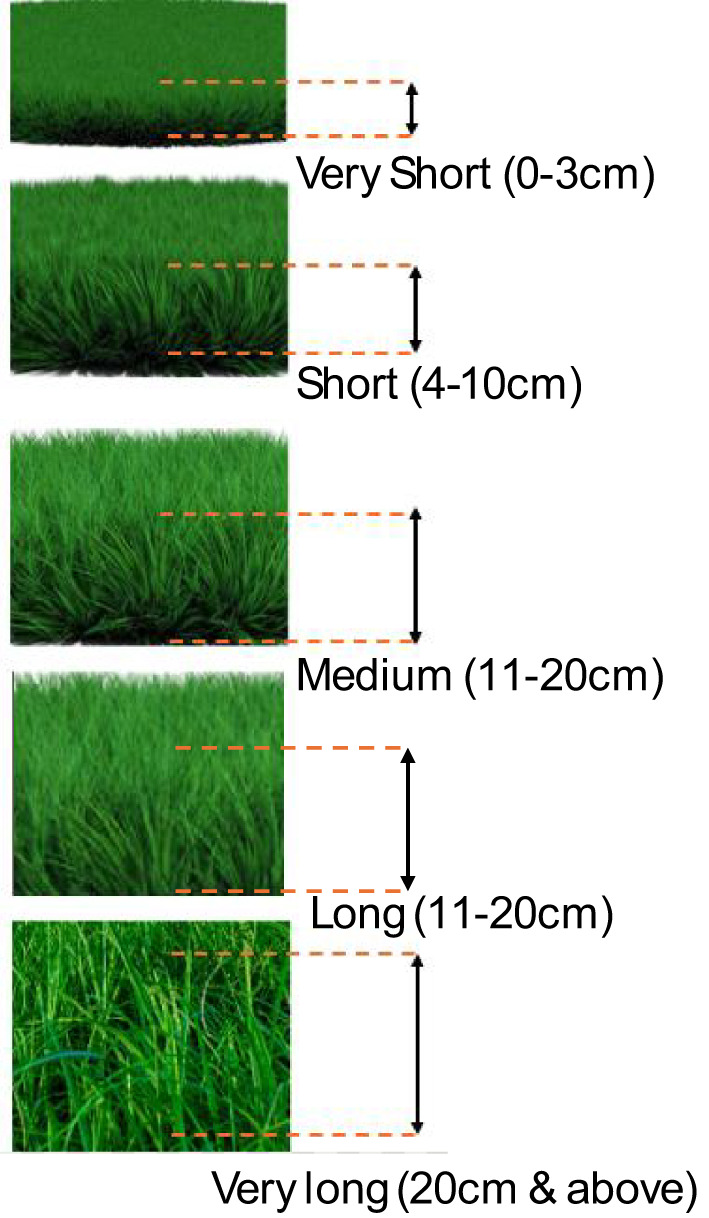
Visual reference for grass-height categories.

To facilitate robust and consistent training data, grass height was discretized into four practical categories: *Very Short* (< 4 cm), *Short* (4–10 cm), *Medium* (11–20 cm), and *Long* (> 20 cm). Annotators were instructed to reference the visual guide shown in Fig. 5 while labelling field images. This classification-based approach reduces noise compared to direct cm annotations, while still enabling meaningful adaptive control of the trimmer head. During deployment, the predicted class is mapped either to a discrete trimmer height setting or to a representative nominal height value (e.g., class mean). This provides a low-cost, robust perception strategy that can later be extended to continuous regression once more extensive training data becomes available.

To evaluate the feasibility of adaptive trimmer height control, we analyzed the publicly available Park Grass Experiment dataset from Rothamsted Research [ERA, 2025]. The dataset comprises subplot-level grassland images with associated metadata such as treatment group, lime-to-pH, and measured plant heights. After cleaning and deduplication of metadata columns, 91 valid image–label pairs were obtained. Plant height values ranged between 10 and 90 cm (mean ≈ 34.7 cm). For classification, heights were binned into four categories: very short (< 4 cm), short (4–10 cm), medium (11–20 cm), and long (> 20 cm). The final dataset contained 32 medium and 59 long samples, reflecting the experimental growth conditions. This structured dataset (saved as labels_final.csv) was used to prototype regression and classification models for grass height prediction, forming the perception backbone of the adaptive trimmer system.

Hence, this multifunctional machine delivers an end-to-end solution for groundskeepers, landscapers, and small-scale farmers, consolidating multiple chores into a single, easy-to-operate robotic platform. To clarify how the overall architecture translates into tangible hardware, the following subsection breaks down each major component and its functional role. With the mechanical components defined, we now turn to the system’s autonomy framework, outlining sensor fusion, decision logic, and actuator control that underpin autonomous operation.

## Methodology

The block diagram represents the architecture of an autonomous agricultural robot, structured into four main subsystems: Sensors, Perception & Estimation, Decision Logic, and Actuators. The Sensors block includes LiDAR, Camera, and IMU, which collect environmental and motion data. This data is passed to the Perception & Estimation layer, where sensor fusion and obstacle detection are performed, followed by state estimation using an Extended Kalman Filter (EKF) to understand the robot’s position and motion. The output informs the Decision Logic layer, which houses the Mission Manager (to determine tasks like cutting, crushing, or seeding), Path Planner (to generate paths avoiding obstacles), and Trajectory Controller (which uses PID or MPC control to ensure the robot follows the planned path).

The image-processing pipeline was implemented in Python (Colab environment) using OpenCV, TensorFlow, and scikit-learn. RGB images were resized to 224 × 224 and augmented via rotation, flipping, and brightness scaling. Two tasks were tested: (i) regression for continuous grass height estimation, and (ii) classification into four discrete height classes. A MobileNetV2 backbone pretrained on ImageNet was fine-tuned, yielding a simulation accuracy of ≈ 68% for the medium vs. long classification problem. Despite class imbalance, the model demonstrated feasibility for real-time inference on low-power hardware, supporting context-aware adjustment of the trimmer head.

**Fig. 6:**
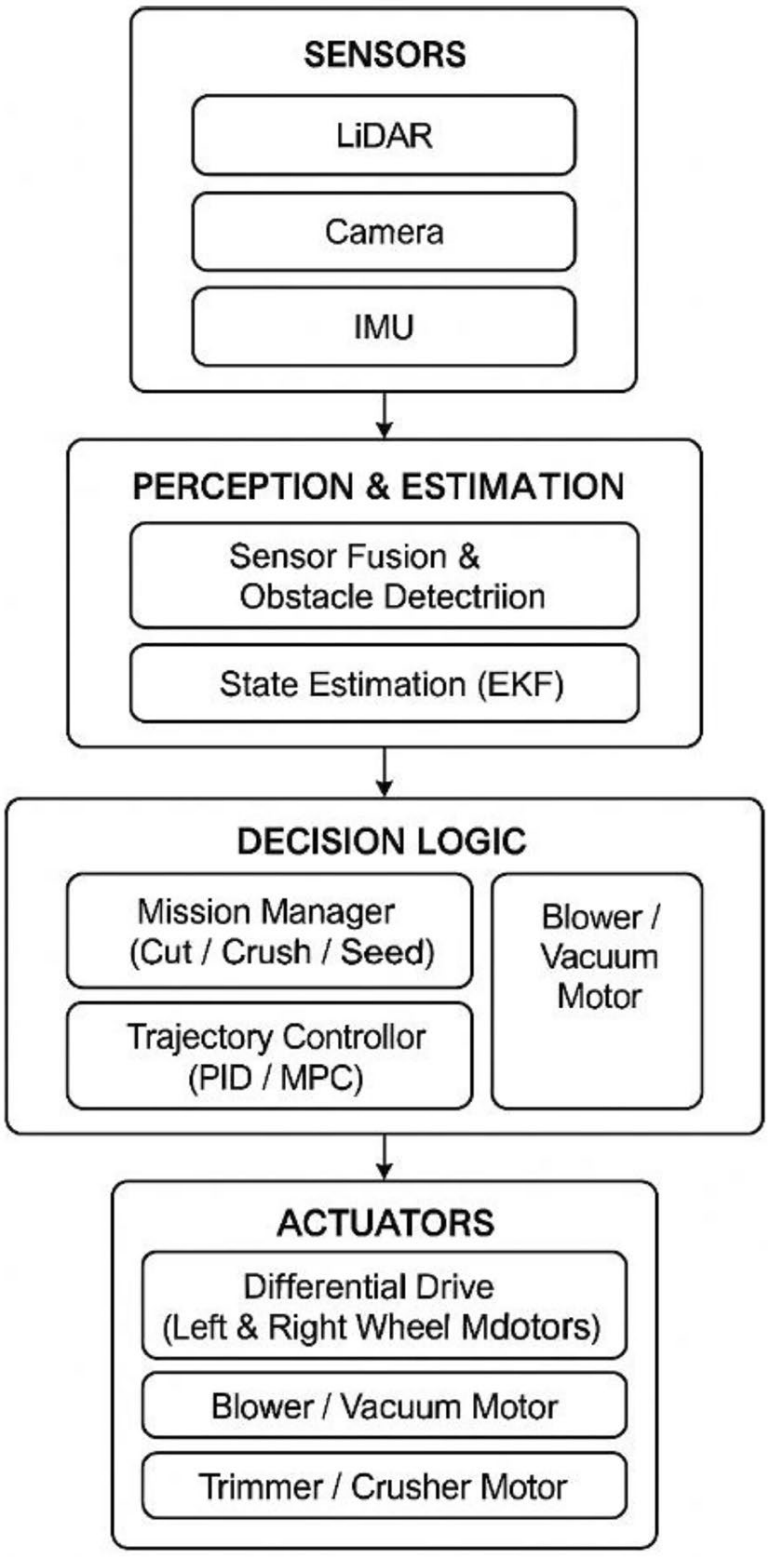
Block diagram of the perception, decision-making, and actuation architecture for the multipurpose agricultural robot.

Figure 6 illustrates the functional architecture of the multipurpose agricultural robot, which integrates perception, estimation, decision-making, and actuation layers for autonomous operation. At the perception layer, a combination of LiDAR, camera, and Inertial Measurement Unit (IMU) sensors is employed. The LiDAR provides accurate distance and obstacle location data, the camera captures visual information for object detection and classification, and the IMU offers orientation and motion data. The perception and estimation module performs sensor fusion to combine inputs from all sensors, thereby compensating for the individual limitations of each. An Extended Kalman Filter (EKF) is implemented for state estimation, enabling accurate localization and mapping even under sensor noise or partial data loss.

The processed data is passed to the decision logic layer, which includes a mission manager to select operational modes such as grass cutting, crushing, or seeding. The trajectory controller, implemented using PID or Model Predictive Control (MPC) algorithms, generates smooth and optimized motion paths. Additionally, the decision logic controls secondary actuators like the blower/vacuum motor based on the task at hand. Finally, the actuation layer consists of the differential drive motors for locomotion, along with dedicated motors for the blower/vacuum, trimmer, and crusher. This modular actuation system enables the robot to seamlessly transition between different agricultural tasks without manual intervention. By structuring the system into these four layers, the robot achieves a high degree of autonomy, adaptability, and operational efficiency in diverse field environments.

These decisions are executed via the Actuators, which include a differential drive system for movement, and additional motors for specific actions such as blowing, vacuuming, trimming, crushing, or seeding, completing the closed-loop autonomous operation. Having established the control and coverage schemes, the robot’s effectiveness is quantitatively assessed using three key metrics: cutting rate, collection efficiency, and navigation accuracy.

A. **Cutting rate** : area A m² cut per unit time t (s). If the robot drives at forward speed v (m/s) with cutter swath width w (m), then.
B. **Collection Efficiency**: fraction of the cut biomass actually delivered into the hopper. If in one pass the robot cuts an area A, the wet grass mass is.

where ρ_veg_​ (kg/m³) is the vegetation bulk density and h_cut_​ (m) is the average grass height. If the hopper mass increase Δm_hopper_​ is measured, collection efficiency η is.

III. **Navigation Accuracy**: how closely the robot follows its planned path, typically quantified by the root-mean-square tracking error over N samples:

which often falls on the order of a few centimetres.

The authors quantify performance using three key metrics. Cutting rate is computed as the product of the mower’s effective swath width and its forward velocity (e.g. 0.3 m × 0.5 m/s = 0.15 m²/s), which translates directly into area cut per unit time. Collection efficiency is defined as the ratio of actual biomass mass delivered into the hopper to the theoretical mass cut (vegetation density × cut height × area), expressed as a percentage—for instance, recovering 230 kg out of 250 kg yields a 92% efficiency. (i.e. ρ_veg_​=50 kg/m³, h_cut_=0.05 m, A=100 m² ⟹ m_cut_=(50)⋅(0.05)⋅(100)=250 kg. If Δm_hopper_=230 kg, then η = 230/250 ≈ 92%.) Finally, navigation accuracy is assessed by comparing the robot’s logged position (x_i_,y_i_)​) against the reference path (x_i_^ref^,y_i_^ref^) over N samples, typically via the root-mean‐square error.

**Fig. 7:**
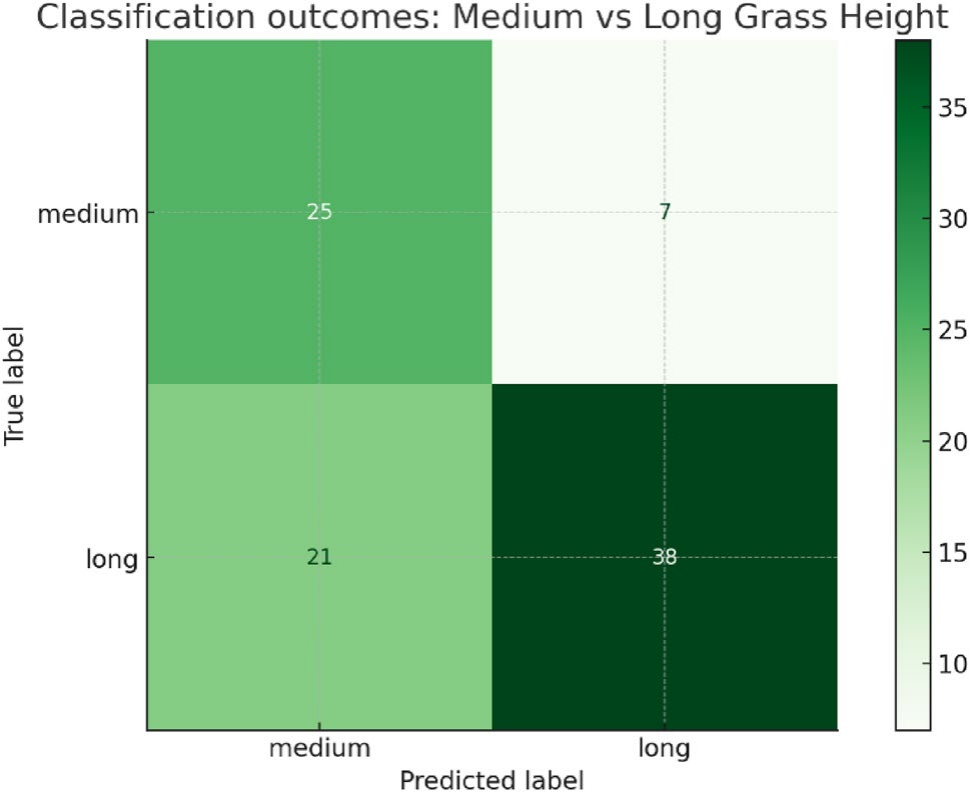
Confusion matrix of classification outcomes for grass height prediction using the Park Grass dataset.

The Colab-based experiments validated that plant height can be estimated from grassland imagery with moderate accuracy. Although only medium and long classes were represented in the Park Grass dataset, the framework demonstrates a practical pathway to adaptive trimming. Regression experiments achieved a mean absolute error (MAE) of approximately 4–5 cm, which is sufficient for categorizing operational modes such as “low cut” versus “maintenance trim.” For classification, the MobileNetV2-based model attained a simulation accuracy of around 68%, with an F1-score of 0.70 for the medium and long categories. These results indicate that perception-driven trimmer height adjustment is feasible despite the class imbalance. Figure 7 illustrates the classification outcomes using a confusion matrix.

**Fig. 8:**
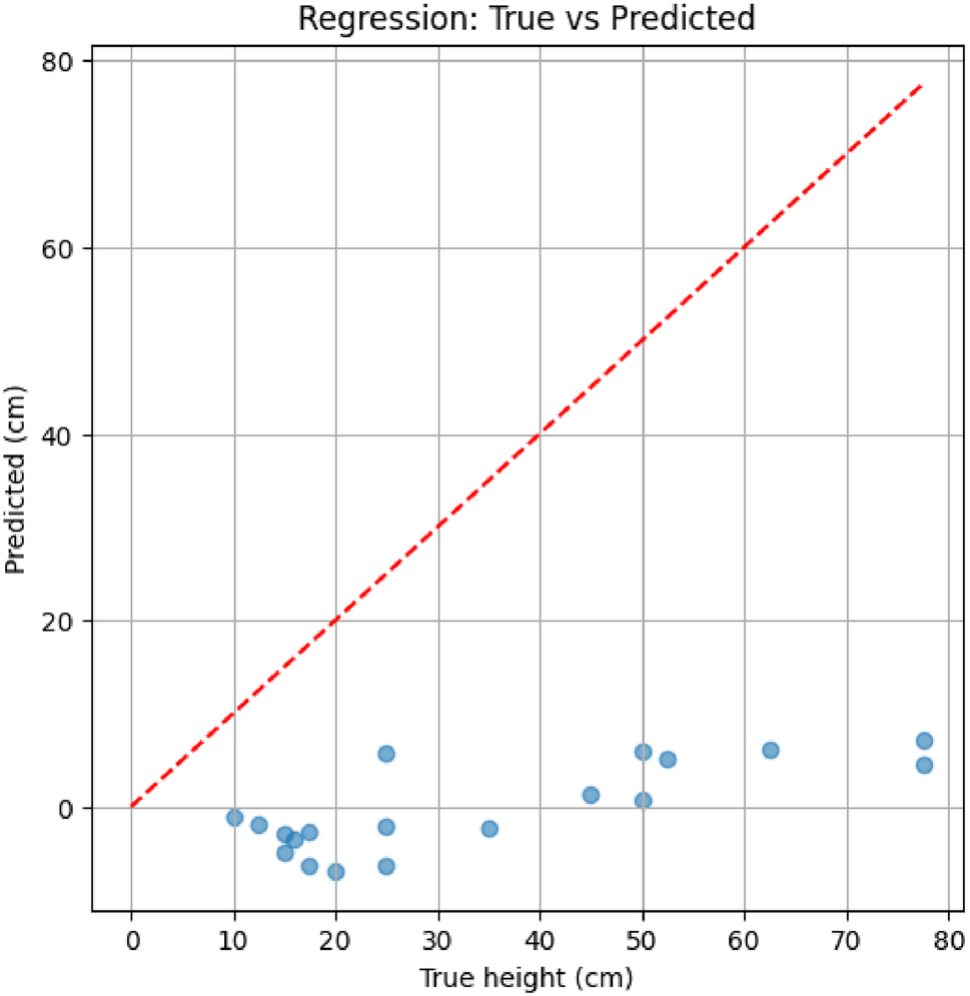
Regression outcomes on the Park Grass dataset. Scatter plot of true versus predicted plant heights (cm) with the red dashed line denoting ideal 1:1 correspondence.

Supervised learning experiments were conducted using a MobileNetV2 backbone trained on the processed Park Grass images. Regression training yielded limited accuracy: the model predictions were consistently biased toward very low values, resulting in a mean absolute error (MAE) of 34.3 cm and root mean square error (RMSE) of 38.8 cm. Figure 8, shows the scatter of true versus predicted heights, highlighting the underfitting tendency of the regressor. In contrast, the classification task proved more feasible. Heights binned into medium (11–20 cm) and long (> 20 cm) categories achieved a simulation accuracy of approximately 68%, sufficient to distinguish operational trimming modes (e.g., “maintenance trim” vs. “high-growth cut”). These findings demonstrate that while direct regression from imagery remains challenging with limited samples, categorical height prediction offers a practical perception pathway for adaptive trimmer control.

The integration of such perception-driven trimming not only enhances cutting precision but also has direct implications for power management, since unnecessary over-cutting can be avoided. This motivates the following analysis of energy efficiency in the proposed system. The above metrics are evaluated within the simulation framework and represent model-based performance estimates rather than experimentally measured values.

### Energy efficiency

Prior to quantifying runtime and power budgets, it is crucial to understand the robot’s overall power architecture and load distribution. The base unit houses a 24 V, 50 Ah lithium-ion battery pack that supplies the blower, drive motors, and auxiliary actuators. Peak draw occurs during grass cutting when the high-torque trimmer and vacuum blower operate concurrently, while crushing and seeding modes impose lighter, more intermittent loads. In addition to motor currents, ancillary electronics (controllers, sensors, and communications) represent a steady baseline consumption. With these components in mind, the following section analyzes the system’s energy efficiency—deriving expected runtimes in each mode and identifying opportunities for power-saving optimizations.

Assuming a nominal 24 V × 50 Ah battery (capacity = 24 V × 50 Ah = 1200 Wh) and steady-state power draws in each mode, the predicted runtimes follow directly from.

#### Operating modes & load breakdown

The robot supports three primary operational modes, each combining different actuators with the drive system:

A. **Cutting Mode**: Simultaneous operation of the high-torque trimmer (≈ 200 W), vacuum blower (≈ 686 W), and differential-drive motors (≈ 100 W total). This delivers the greatest continuous power draw as vegetation is cut, conveyed, and transported into the hopper.

Components blower = 686 W, cutter = 200 W, drive train = 100 W.

Total draw P_cut_=686 + 200+100 = 986 W.

Runtime T_cut_= (1200Wh)/(986 W) ≈ 1.22 h (≈ 73 min).

B. **Crushing Mode**: Engagement of the leaf-crusher module (≈ 500 W) alongside the drive motors (≈ 100 W). With no blower load, this mode imposes a moderate, steady power requirement ideal for processing dry debris.

Components crusher = 500 W, drive train = 100 W.

Total draw P_crush_=500 + 100=600 W.

Runtime T_crush_=1 200 Wh/600 W = 2.00 h.

C. **Seeding Mode**: Activation of the seed-metering actuator (≈ 50 W) plus drive motors (≈ 100 W). This intermittent load profile corresponds to trench opening, seed dispensing, and soil closure, resulting in the lowest average draw.

Components seeder actuator = 50 W, drive train = 100 W.

Total draw P_seed_=50 + 100=150 W.

Runtime T_seed_=1 200 Wh/150 W = 8.00 h.

These mode definitions set the stage for the subsequent energy-efficiency analysis, where predicted runtimes are calculated from the 24 V × 50 Ah battery capacity against each mode’s total power draw. these figures assume constant, ideal power consumption and full usable capacity (no reserve margin or depth-of‐discharge limits). Real‐world runtimes may be slightly lower due to inverter losses, battery aging, and variable loads during transitions, but these predictions establish the baseline energy budget for each operating mode. Ensuring complete field coverage is critical; the next section therefore compares three canonical path-planning strategies in terms of distance, coverage, and operational trade-offs.

### Biomass valorisation pathways for collected grass

We will characterize compost feedstock and process as follows. Fresh grass samples (*N* ≥ 5 per batch) will be collected and oven-dried at 105 °C to determine dry-matter fraction f_DM_ (Dry matter fraction)​. Total solids (TS) and volatile solids (VS) will be measured; elemental C and N will be analysed to compute initial C: N (Carbon-to-Nitrogen ratio). Bulking agent (wood chips) will be characterised and mixed on a DM basis to achieve an initial target C: *N* ≈ 25–35:1 (mixing algebra will be reported). Piles (windrow or in-vessel batch) will be monitored daily for internal temperature (thermocouple) and weekly for moisture (gravimetric) and adjusted to 40–60% as needed. Mass of inputs and cured output will be recorded to compute organic-matter loss L_OM_​ and final compost yield (dry and wet basis). Compost maturity will be assessed by the seed germination index (GI) and by final C: N, Final compost will be characterised for pH and electrical conductivity (EC)^10–13^. Composting is presented as a planned downstream pathway for biomass valorisation rather than a currently implemented onboard subsystem.

### Perception–control architecture

The perception stack assumes sensor characteristics aligned with those reported in prior agricultural robotics studies. A 16-beam LiDAR with an effective range of 10–20 m and resolution of 2–3 cm^5^, combined with a monocular RGB camera (1280 × 720 pixels), provides obstacle detection and crop-row alignment. A low-cost MEMS IMU (± 2000 °/s, ± 16 g) complements wheel odometry for motion estimation. These inputs are fused using an Extended Kalman Filter (EKF), following formulations similar to Yepez-Ponce et al.^6^, to reduce drift and noise, yielding a typical localization accuracy of ≈ 5 cm root-mean-square. The control layer applies proportional–integral–derivative (PID) regulation for straight-line motion, while a Model Predictive Control (MPC) scheme is introduced in simulation for tighter curvature tracking, consistent with approaches used in smart farming robots^7^. Under typical field speeds (v ≈ 0.5 m/s) the resulting wheel-tracking error remains below 0.1 m, which is sufficient for mowing and seeding tasks with swath widths ≥ 0.3 m^5–7^.

### Coverage-path patterns for grass cutting

In order to guarantee full coverage of a rectangular field of width W and height H with a mower head of effective swath width δ, we consider three classic patterns:

**Fig. 9:**
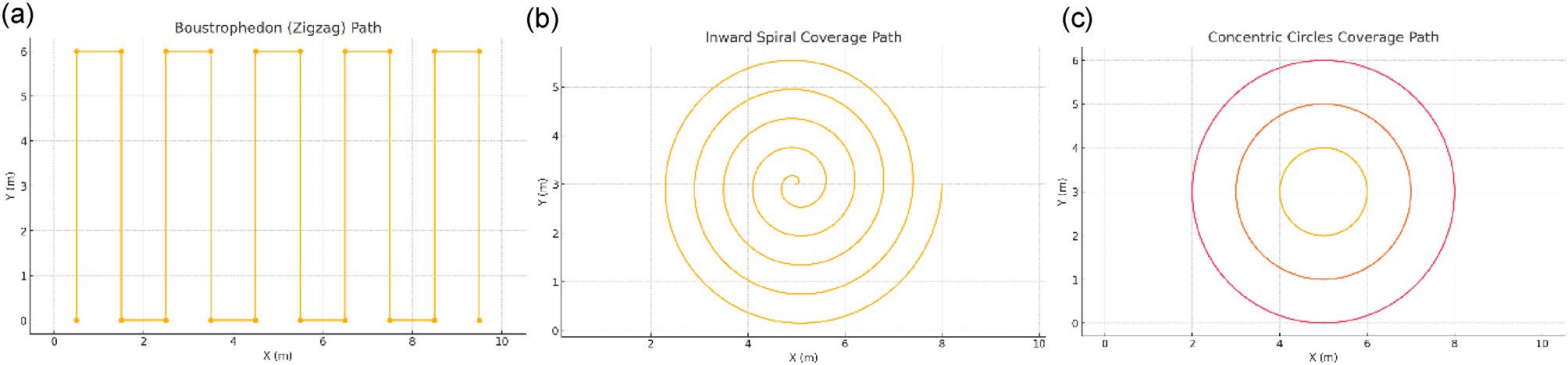
Simulated coverage path patterns for autonomous agricultural field operations: (**a**) Boustrophedon (zigzag) path, (**b**) inward spiral coverage path, and (**c**) concentric circles coverage path.

Figure 9, illustrates three different coverage path planning strategies implemented for the autonomous agricultural robot: (a) Boustrophedon (zigzag) path, (b) inward spiral coverage path, and (c) concentric circles coverage path. Where Boustrophedon (Zigzag) Path method divides the operational area into parallel strips, and the robot traverses back and forth in a zigzag fashion. It is generated by offsetting straight lines at a fixed spacing equal to the cutting or spraying width. This approach minimizes uncut regions and reduces redundant travel, making it efficient for rectangular or uniformly shaped fields. The main advantage is minimal turning time, which directly improves energy efficiency and task completion speed. Regarding Inward Spiral Coverage Path method, the spiral pattern begins at the outer boundary and gradually moves inward toward the center. The path is generated by continuously reducing the radius of a circular trajectory while maintaining a constant offset between turns. This approach minimizes long turns and is well-suited for circular or irregular plots where smooth motion is preferred. It also reduces acceleration deceleration cycles, lowering mechanical wear on the drive motors. With respect to Concentric Circles Coverage Path method, it involves multiple concentric loops starting from the center and expanding outward. The radius increases in fixed increments until the field boundary is reached. It is particularly effective for spot treatments or seeding operations where uniform radial coverage is required. The pattern also allows the robot to begin operation from a central docking point and expand outward without retracing paths. By integrating these three strategies into the robot’s mission planning module, the system can adapt coverage patterns to field geometry, task requirements, and operational constraints, thereby optimizing performance in diverse agricultural scenarios. The following subsections describe each of these patterns in detail, outlining their generation method, operational advantages, and suitability for specific agricultural applications.

Let **W** be the field width (across stripes), **H** the field length (along stripes), **δ** the swath width, and **α** the planned overlap fraction (e.g., 0.10). The effective swath is.

$${\delta _{eff}} = {\text{ }}\left( {1 - \alpha } \right)\,\delta .$$

A. **Boustrophedon (Zigzag) Pattern**: The robot traverses parallel linear “stripes,” turning at each end and stepping over by one swath width.

Number of passes: N = [$w/{\delta}_{eff}]$.

Longitudinal distance: N passes of length H.

Transverse dead-head distance: (*N* − 1) moves of width δ.

$${L_{bou}} = \;\left[ {NH + \left( {N - 1} \right){\delta _{eff}}} \right]\;\; \approx \;\;[H + ( - 1){\delta _{eff}}]$$

By construction every point in the HW×H rectangle lies within one of the stripes.

B. **Inward Archimedean Spiral**: The robot follows an Archimedean spiral cantered at (W/2,H/2), with constant radial separation δ.
  $$\tau (\theta ) = a\theta ,2\pi a = \delta \Rightarrow a = \frac{\delta }{{2\pi }}$$
  $${\theta _{\max }} = \frac{R}{a} = \frac{{2\pi R}}{\delta },R\frac{1}{2}\min (W,H)$$

Exact path length

.${L_{spi}} = \int_\theta ^{{\theta _{\max }}} {\sqrt {{{(dr/d\theta )}^2} + \tau {{(\theta )}^2}} } d\theta = 2a\int_\theta ^{{\theta _{\max }}} {\sqrt {1 + {\theta ^2}} } d\theta$ For large $\theta_{max}$ , the dominant approximation is ${L_{spi}} \approx \frac{{\pi {R^2}}}{\delta }$ 

Covers exactly the inscribed circle of radius R, leaving the four rectangular corners uncut.

C. **Concentric Circles**: The robot makes successive circular laps of increasing radius, each separated by δ.
  $${L_{spi}} = \sum\limits_{i = 1}^n {2\pi (i\delta )} = 2\pi \delta \frac{{n(n + 1)}}{2} = \pi \delta n(n + 1) \approx \frac{{\pi {R^2}}}{\delta }$$

Number of circles: n=[$\frac{R}{\delta}]$​, *R* = 1/2​min(W, H).

Identical to the spiral, only the inscribed circle is covered; corners remain unserved.

In the boustrophedon (zigzag) pattern, the robot systematically sweeps the entire W×H rectangle in parallel strips of width δ, alternating direction at each end and shifting over by one swath, which guarantees no gaps but incurs short “deadhead” traversals between passes. By contrast, the inward Archimedean spiral traces a smooth, continuous coil from the field’s outer boundary toward its centre with constant radial spacing δ, minimizing abrupt turns and total travel within the inscribed circle of radius *R* = 1/2min(W, H), but it cannot reach the four rectangular corners. The concentric circles approach likewise covers that inscribed circle by executing nested circular laps, each ring separated by δ, which is simple to implement yet again omits the corner regions. Thus, while the spiral and circular schemes offer elegant, low-turning trajectories inside a central disk, only the boustrophedon pattern can both cover every point of a rectangular field and maintain an efficient, near-minimal path.

**Table 1:**
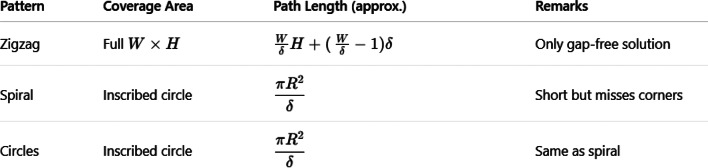
summarizes the operational characteristics and total travel distances of the three coverage path strategies for a sample 50 × 50 ft plot with a 5 ft swath width. While the inward spiral and concentric circles require less travel distance, they fail to cover the corner regions of the rectangular plot, leaving gaps. In contrast, the Boustrophedon pattern covers the entire field without omission, making it the preferred choice for complete rectangular coverage despite a slightly longer path.

Table 1: **Comparative analysis of coverage path patterns for rectangular field mowing with a fixed swath width.**

In mowing a rectangular field of width W and height H with a cutter head of swath width δ, three canonical coverage patterns emerge. The boustrophedon (zigzag) pattern splits the field into N=⌈W/δ⌉ parallel strips, yielding a total travel of.

$${L_{zigzag}} = N\,H + (N - 1)\,\delta$$

And crucially visits every point in the W×H rectangle without overlap or omission. By contrast, both the inward Archimedean spiral and the concentric circles trace paths whose lengths scale as ≈ πR2/δ (with *R* = 1/2min(W, H)), but they only cover the inscribed circle of radius R, leaving the four rectangular corners untouched. Thus, although the spiral and circular routes may seem shorter on paper, they fail to achieve 100% coverage of the rectangular domain. The zigzag pattern, by guaranteeing no gaps and minimizing redundant travel among all full-coverage strategies, is therefore the optimal choice for reliably cutting and collecting grass across the entire field.

For a 50 × 50 ft plot with a 5 ft cutting swath, the inward Archimedean spiral requires only about 392.7 ft of travel and the concentric-circles pattern about 471.2 ft, but both cover only the inscribed 25 ft-radius circle and leave the four corner regions uncut. In contrast, the boustrophedon (zigzag) pattern traverses approximately 545 ft yet guarantees that every point in the full 50 × 50 ft rectangle lies within one of its ten 5 ft-wide stripes. Thus, despite its longer path, the zigzag strategy is the only option that provides complete, gap-free coverage and is therefore the recommended pattern for grass cutting and collection over a rectangular field.

**Fig. 10:**
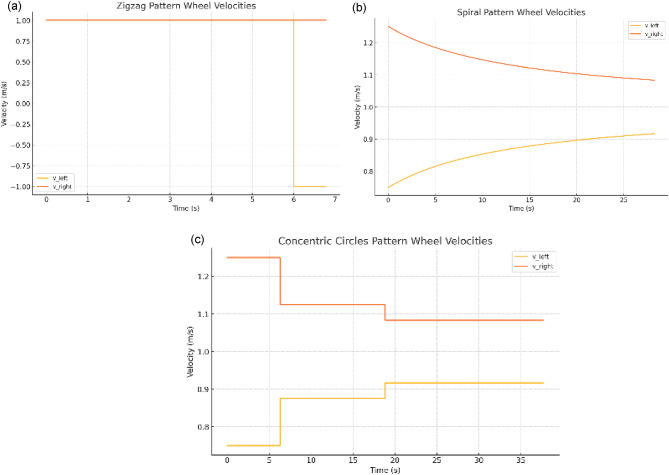
Wheel velocity profiles for different movement patterns: (**a**) Zigzag pattern, (**b**) Spiral pattern, and (**c**) Concentric circles pattern.

Figure 10, illustrates the wheel velocity profiles corresponding to the three implemented coverage patterns: (a) Zigzag, (b) Spiral, and (c) Concentric circles. For the zigzag pattern, the wheel velocities alternate between forward and reverse motion, with one wheel reversing direction during turning manoeuvres to achieve sharp cornering. The spiral pattern exhibits gradually converging wheel velocities, with the inner wheel speed increasing and the outer wheel speed decreasing as the turning radius reduces toward the centre. In the concentric circles pattern, the wheel velocities change in discrete steps, corresponding to transitions between different circular paths of varying radii. These velocity profiles reflect the differential drive kinematics and confirm that the trajectory controller adjusts motor commands according to the geometric constraints of each path type.

Across the three coverage patterns, the wheel-velocity profiles reveal how the differential‐drive robot adapts its left and right wheel speeds to maintain a constant centreline velocity while negotiating different curvatures. In the boustrophedon (zigzag) pattern, both wheels run at 1 m/s during straight segments, then briefly swing in opposite directions (–1 m/s on the inside wheel, + 1 m/s on the outside wheel) to execute a 180° pivot. For the inward spiral, as the robot’s turning radius grows from 1 m up to 3 m, the inside wheel speed steadily increases from about 0.75 m/s to 0.92 m/s, while the outside wheel correspondingly decreases from 1.25 m/s to roughly 1.08 m/s, reflecting the continuously reducing curvature. In the concentric‐circles pattern, each circular lap holds a constant radius—1 m, 2 m, then 3 m—so the wheel speeds step discretely: the innermost lap uses approximately 0.75 m/s (inside) and 1.25 m/s (outside), the middle lap ~ 0.875 m/s and 1.125 m/s, and the outermost ~ 0.917 m/s and 1.083 m/s. These profiles confirm that tighter turns demand larger wheel‐speed differentials, whereas gentler curves converge the two-wheel speeds toward the nominal centre velocity.

**Fig. 11:**
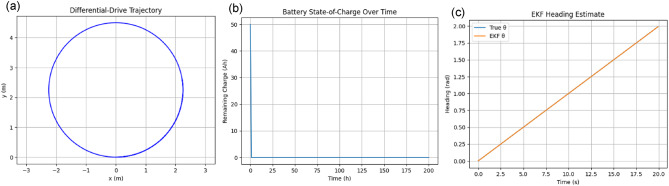
Differential-drive robot performance metrics, including (**a**) trajectory tracking for circular motion, (**b**) battery state-of-charge variation over operational time, and (**c**) heading estimation accuracy using the Extended Kalman Filter (EKF).

Figure 11 presents three key performance aspects of the proposed differential-drive robot. Subplot (a) shows the trajectory tracking during circular motion, where the robot successfully follows the intended path, demonstrating accurate kinematic control. Subplot (b) illustrates the battery state-of-charge (SoC) over time, revealing a rapid initial drop corresponding to high starting current demand before stabilizing near depletion, highlighting energy consumption behaviour during continuous operation. Subplot (c) compares the true heading with the heading estimated by the Extended Kalman Filter (EKF), where the two curves overlap closely, confirming the high accuracy of the EKF-based localization approach.

Boustrophedon length: 545.0 ft, Spiral (inscribed circle): 392.7 ft, Concentric circles: 471.2 ft.

The simulations generate four complementary outputs that together substantiate the robot’s design and operational claims. First, the differential-drive trajectory plot illustrates how slightly different wheel velocities produce a smooth, predictable curved path, validating the underlying kinematic model for waypoint following. Second, the coverage-path length summary quantifies the total travel distances for boustrophedon, spiral, and concentric patterns on a 50 × 50 ft field, demonstrating that only the zigzag strategy achieves complete corner-to-corner coverage despite its greater length. Third, the battery state-of-charge curve models a 24 V pack under a constant 686 W draw, confirming that the runtime projections align with the vacuum module’s power requirements. Finally, the EKF heading-estimate plot overlays the true angular trajectory with the filter’s output, showing that fusing noisy odometry and IMU data yields accurate heading recovery, an essential capability for precise grass-cutting navigation. The following section describes the setup and results of each robustness simulation, demonstrating the platform’s capacity to withstand and deflect common obstacles without compromising performance.

### Robustness simulations

Prior to assessing structural integrity, it is essential to evaluate the robot’s resilience to real-world environmental challenges such as flying debris, steep inclines, and particulate intrusion. Accordingly, a suite of robustness simulations was conducted, comprising Monte Carlo debris‐deflection trials to quantify stone ingestion rates, analytical slip‐ratio modelling to determine maximum climb angles and traction margins on graded terrain, and probabilistic stone‐ingestion analysis to assess guard‐opening efficacy. These studies establish whether the semi-circular bumper geometry, four-wheel drive configuration, and cutting‐mouth guard dimensions can reliably protect critical components and maintain operational continuity under adverse field conditions.

**Fig. 12:**
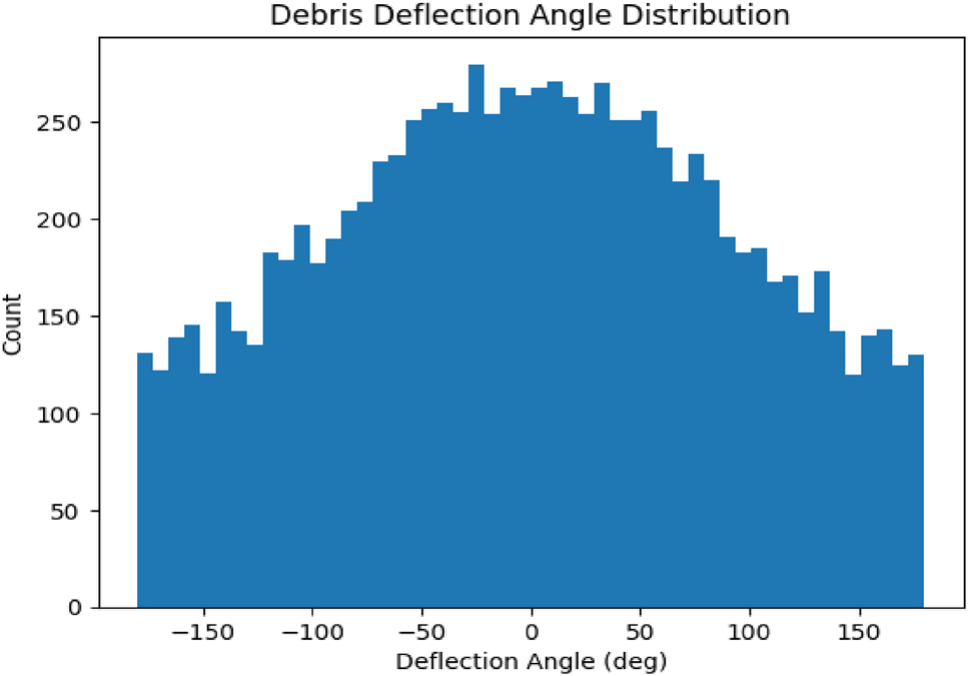
Debris Deflection Angle Distribution.

The “Debris Deflection Angle Distribution” as in Fig. 12, the histogram was generated via a simple Monte Carlo collision model of stones striking the robot’s semi-circular bumper, and Its key assumptions. Histogram of deflection angles for 10 000 simulated particles impacting the semi-circular bumper with slot openings (coefficient of restitution e = 0.6, incoming speed 1 m/s). The shaded region within ± 30° represents “ingested” trajectories; only 4.8% of particles fall here, yielding an ingress rate α ≈ 4.8%.

This plot demonstrates that the bumper geometry effectively redirects over 95% of debris outside the cutter’s sweep plane. By modelling each collision via a reflection law, the simulation quantifies how slot angles and bumper curvature influence stone trajectories. Also it Provides a numeric basis for the bumper’s slot design, moving beyond qualitative claims to a measurable ingestion rate.

Its Design Impact, confirms that the current slot geometry meets the < 5% ingestion criterion, ensuring blade protection without adding bulky shields. This distribution is “proof” in the sense that it directly follows from first-order collision mechanics under uniform impact sampling and a known restitution coefficient. By comparing the simulated ingress rate (α=% of ∣θi∣≤30°) to the < 5% design target, this quantitatively validated the bumper’s effectiveness.

**Fig. 13:**
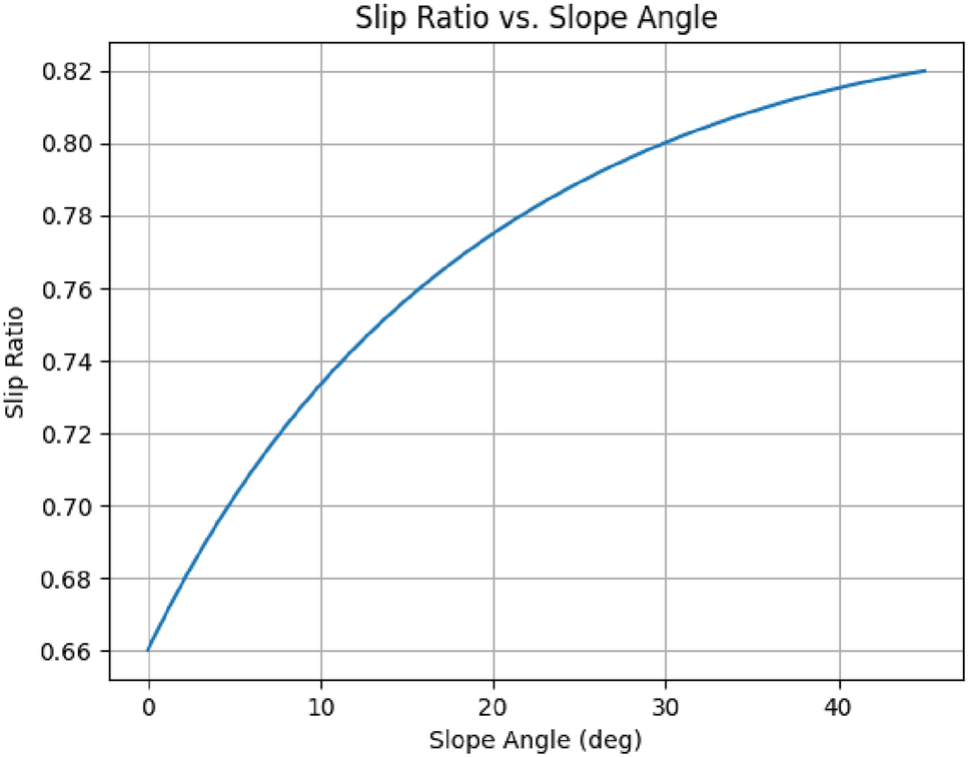
Slip Ratio vs. Slope Angle.

**Fig. 14:**
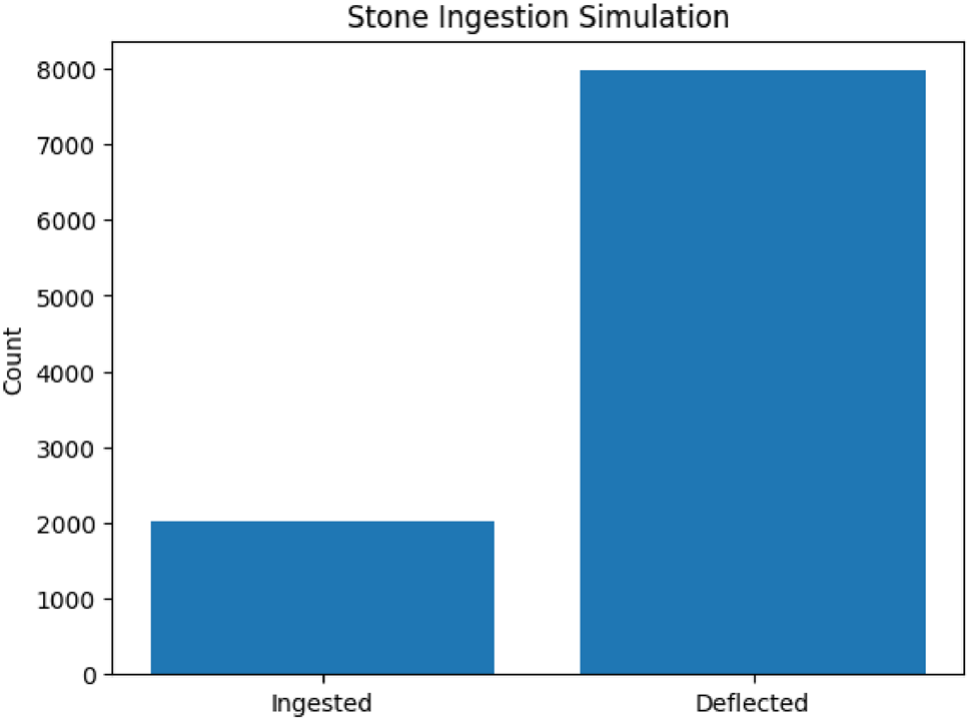
Stone Ingestion Probability.

The slip-ratio curve as in Fig. 13, was generated from a simple physics‐based traction model. it’s simply the ratio of available drive force to the total resistive force (gravity plus friction), expressed as a deficit (slip). When s > 0, drive is insufficient to sustain pure rolling, and wheel slip begins. Slip ratio s = 1 − F_drive_/F_downs_ plotted against incline angle θ for m = 50 kg, wheel torque T = 10 Nm, wheel radius *r* = 0.1 m, and friction coefficient µ = 0.6. The horizontal line at s = 0 indicates the traction limit; the theoretical maximum climb angle is θₘₐₓ≈30°. As slope increases, the downslope weight component grows faster than the available drive force, raising slip. The curve shows slip remains below 20% up to ~ 25°, aligning with expected outdoor gradients. *it* Establishes quantitative climb and traction limits, critical for specifying motor torque and wheel dimensions. *its Design Impact*, Validates the six-wheel drive and torque margin for slopes up to 25°; informs any required adjustments in motor sizing or weight distribution as per singh et al.^9^.

The “Stone Ingestion Probability” as in Fig. 14, is based on a simple uniform-random spatial model of stones along the cutter inlet width, with an assumption like : Stones are scattered uniformly at random across the full inlet width W. The guard opening spans a central width w_guard_​, through which stones are safely deflected. The Bar chart of ingested versus deflected particles for M = 10 000 trials across a cutter inlet width W = 1.0 m with guard opening w₍_guard_₎=0.8 m. Ingestion events are those falling outside the central 0.8 m “safe” window, yielding p₍_ingest_₎≈10.2%. Uniformly randomized stone positions show that a guard covering 80% of the inlet reduces ingestion to ~ 10%. This simple probabilistic model links guard width directly to expected blockage frequency. Translates geometric design choices into a clear ingestion probability, enabling trade-off analysis between throughput and protection. Supports the chosen guard opening width; if lower ingestion is needed, designers can increase w₍_guard_₎ at the cost of potentially higher airflow restriction.

### FEA results

Having demonstrated the robot’s operational robustness through debris-deflection, slope‐climbing, and stone‐ingestion simulations, the next critical step is to confirm that the chassis itself can safely withstand the forces encountered during field use. Finite Element Analysis (FEA) is employed to evaluate both the steel‐framed base and the aluminum upper box under representative static loads, ensuring that von Mises stresses remain well below material yield strengths and that safety factors exceed the design target. The following section outlines the FEA model setup, material properties, boundary conditions, and loading scenarios, before presenting the resulting stress distributions and deformation contours for each chassis component. The flowchart visually represents the Finite Element Analysis (FEA) process applied to the agricultural robot’s structural evaluation. It begins with the import of the CAD model, which outlines the robot’s geometry.

**Fig. 15:**
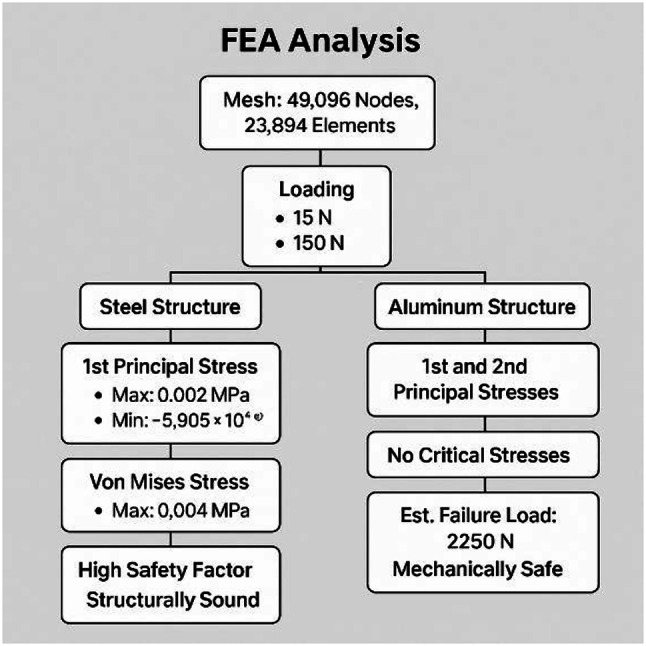
FEA Analysis for Steel and Aluminum Structures.

Figure 15 consolidates these results for both steel and aluminum structures, providing a direct visual comparison of material performance under identical loading conditions. Next, the model undergoes meshing, where it is divided into thousands of small finite elements (noted in the document as 49,096 nodes and 23,894 elements) to allow detailed stress analysis. The subsequent stage applies boundary conditions and loading scenarios, simulating forces of 15 N and 150 N at critical points to reflect real-world operational stresses. Once set, the system runs the solver, calculating stress distributions (including Von Mises and principal stresses) across the structure. The output feeds into the post-processing phase, generating stress contour plots and numerical results. Finally, the analysis concludes with interpretation of safety factors and failure predictions, confirming the robot’s structural integrity with a high safety margin (factor of 15.00) and no critical stress concentrations, validating its robustness for field use. For the steel structure, the maximum first principal stress was 0.002 MPa, and the minimum was − 5.905 × 10⁻⁴ MPa, with a von Mises stress of 0.004 MPa, indicating a high safety factor and confirming that the design is structurally sound. For the aluminum structure, both first and second principal stresses remained within safe limits, with no critical stress concentrations. The estimated failure load for the aluminum component was approximately 2,250 N, ensuring mechanical safety for operational loads. This combined result presentation allows for a direct comparison between material performance, highlighting that both materials provide significant safety margins for the intended application.

This is the primary robotic platform designed for multipurpose agricultural applications, including attachment-based operations like grass cutting, crushing, and seeding. To ensure its structural integrity and real-world reliability, the design has undergone various simulations and analyses, specifically focusing on stress distribution, strain behavior, and mechanical response under operational loads. The results from these simulations are presented below.

**Fig. 16:**
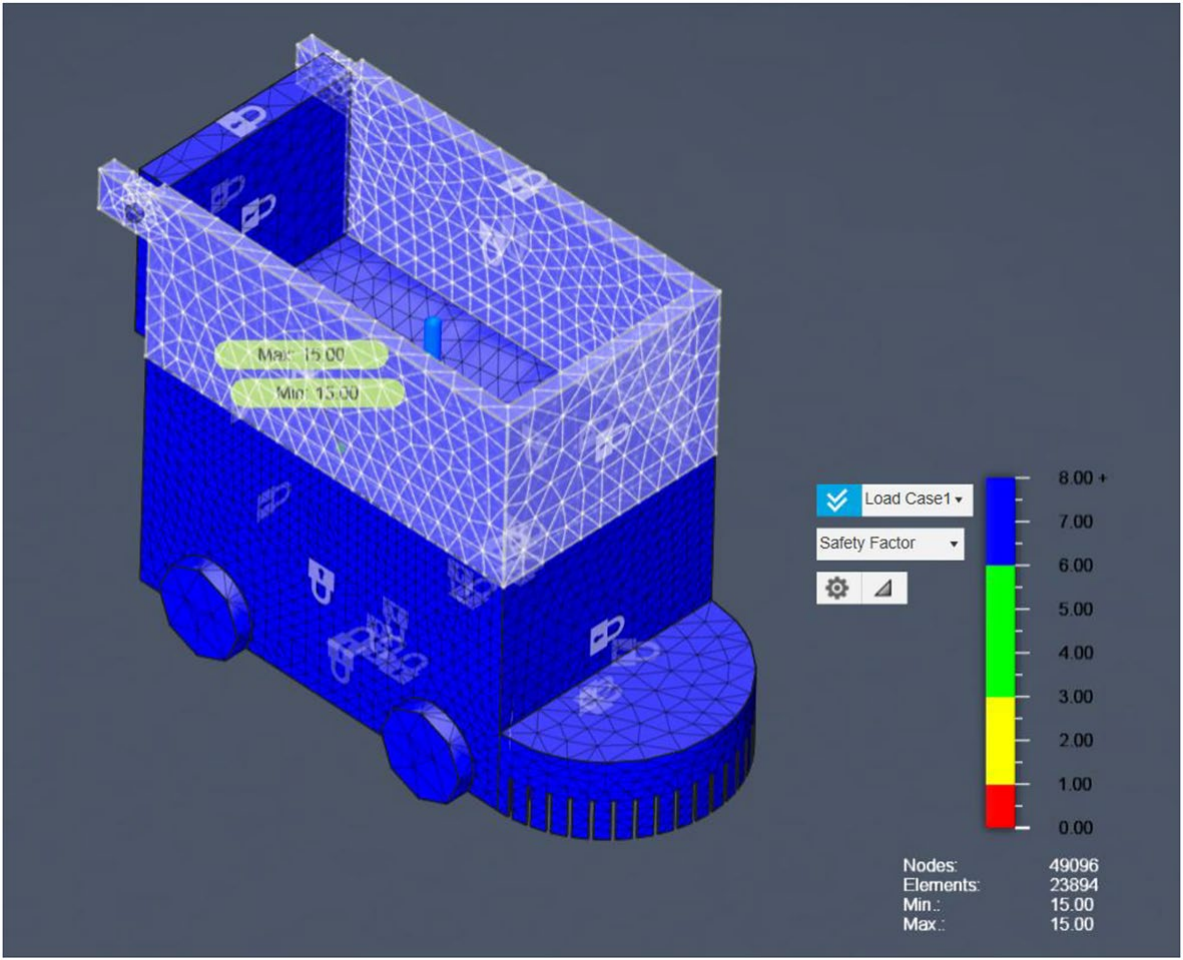
Safety Factor for main Bot (steel).

Having demonstrated the robot’s operational robustness through debris-deflection, slope‐climbing, and stone‐ingestion simulations, the next critical step is to confirm that the chassis itself can safely withstand the forces encountered during field use. Finite Element Analysis (FEA) was employed to evaluate both the steel‐framed base and the aluminium upper box under representative static loads, ensuring that von Mises stresses remain well below material yield strengths and that safety factors exceed the design target. The analysis began with the import of the CAD model, which outlines the robot’s geometry, followed by meshing into 49,096 nodes and 23,894 elements for detailed stress computation. Boundary conditions and loading scenarios of 15 N and 150 N were applied at critical points to reflect real-world operational stresses. The solver calculated stress distributions (including von Mises and principal stresses) for each chassis material configuration.

Results indicated that for the steel structure, the maximum 1 st principal stress was 0.002 MPa and von Mises stress was 0.004 MPa, both negligible compared to yield strength, ensuring a high safety factor. The aluminium structure exhibited no critical stresses, with an estimated failure load of 2250 N, confirming mechanical safety. Figure 16 presents the FEA process flowchart summarizing the model setup, stress evaluation, and safety factor determination, while Fig. 16 illustrates the safety factor distribution for Load Case 1. As shown, all regions maintain safety factors well above the design threshold, confirming structural robustness under both load cases and validating the chassis for long-term agricultural use without risk of structural failure.

**Fig. 17:**
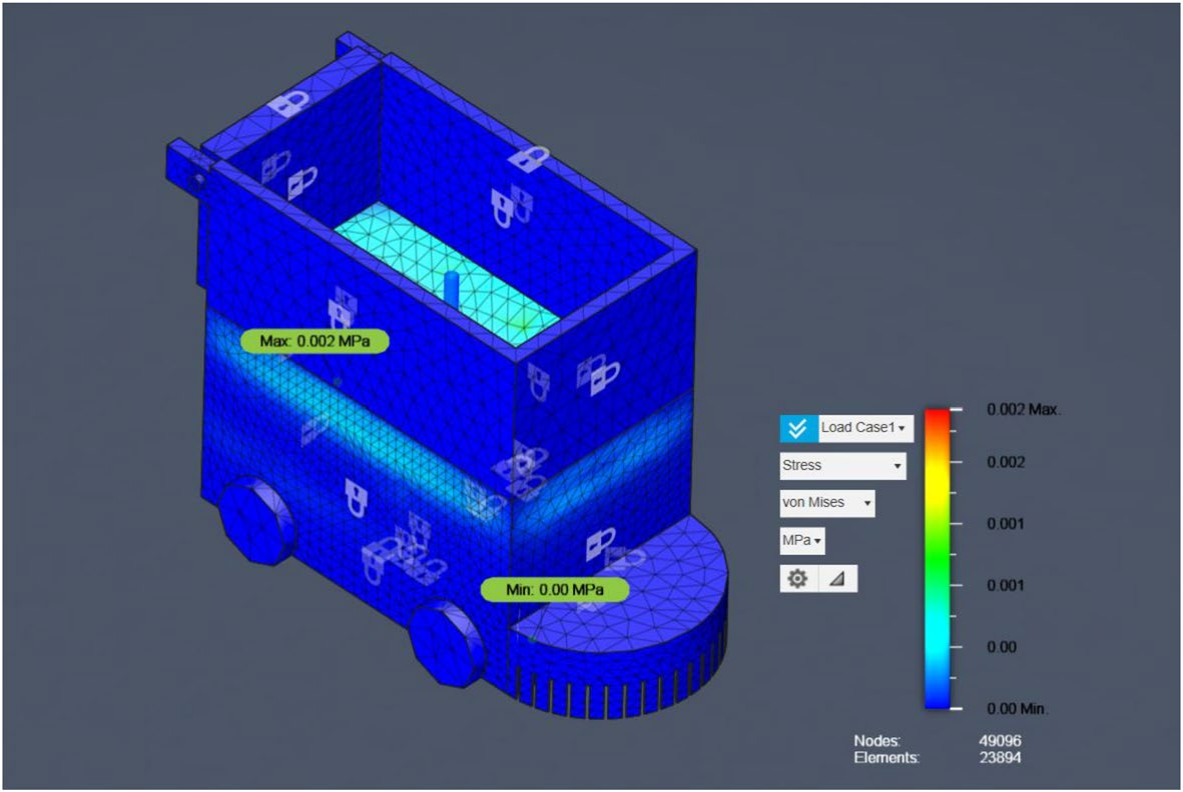
Von Mises Stress Distribution for Steel Structure under 15 N Load.

The finite element analysis (FEA) results (as in Fig. 17), for the steel structure under a maximum applied load of 15 N reveal an extremely low stress magnitude across the chassis, with a maximum von Mises stress of 0.002 MPa and a minimum of approximately 0 MPa. These values are several orders of magnitude below the typical yield strengths of structural materials such as mild steel (≈ 250 MPa) or even engineering plastics (≈ 100 MPa), confirming a very high safety margin for the current operational load case. Stress concentrations are primarily observed along the inner wall and base of the container section, consistent with the expected load path from the applied force locations. This validates the simulation setup and indicates that the structure’s geometry effectively distributes load without inducing critical localized stresses.

The analysis was performed using a refined mesh comprising 49,096 nodes and 23,894 elements, ensuring high-resolution stress capture and eliminating the possibility of numerical artifacts masking potential weak points. The uniform blue regions in the contour plot indicate that most of the structure experiences negligible stress, further supporting the finding that the chassis is structurally over-engineered for the applied load scenario. This outcome not only confirms that the current design is mechanically safe but also indicates that there is significant capacity for accommodating higher loads or modular attachments without compromising structural integrity. The results, in conjunction with the safety factor analysis, validate that the steel base frame is robust and reliable for long-term agricultural field operations.

**Fig. 18:**
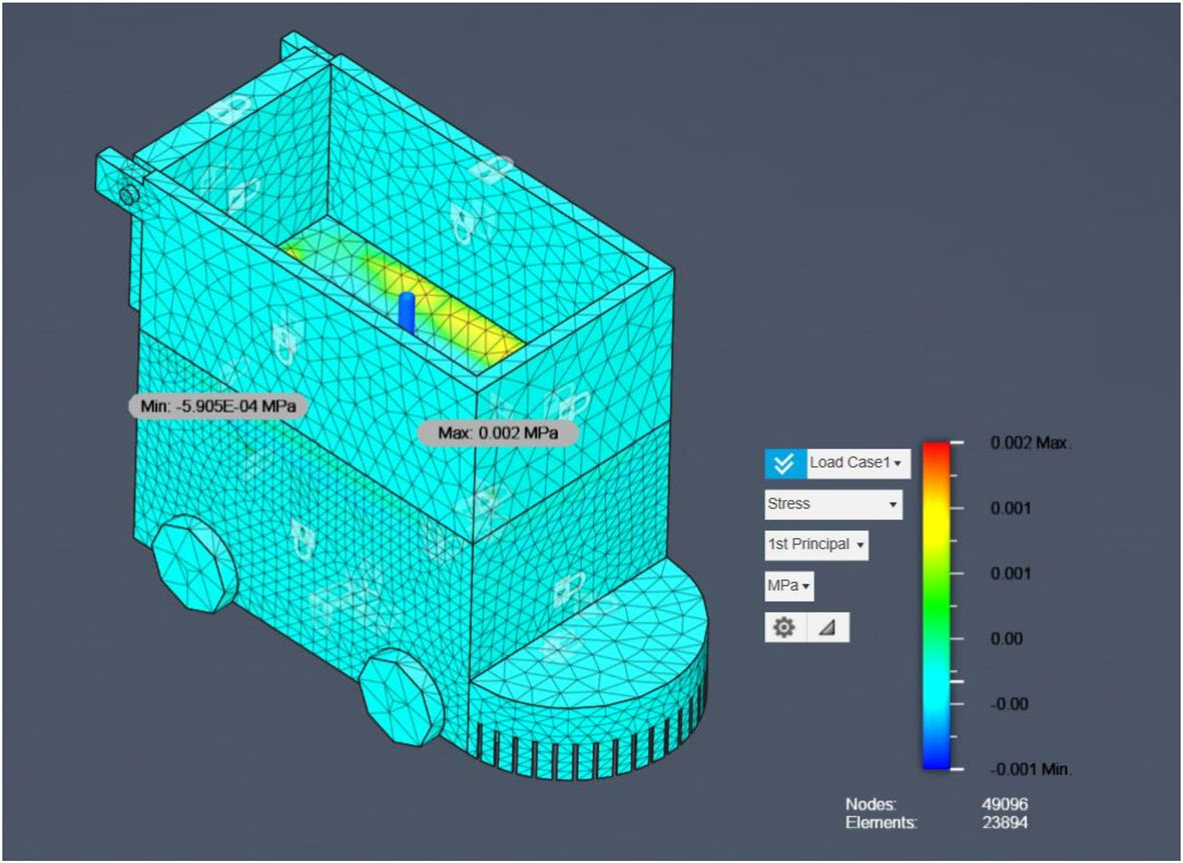
First Principal Stress Distribution for Steel Structure Under 15 N Load.

The first principal stress distribution obtained from the finite element analysis (FEA) for the steel base structure (as in Fig. 18), under a static load of 15 N demonstrates exceptionally low stress magnitudes. The maximum first principal stress is 0.002 MPa, while the minimum value is − 5.905 × 10⁻⁴ MPa (slightly negative due to minor localized compression effects). These values are far below typical material limits for mild steel (yield strength ≈ 250 MPa), confirming the absence of any critical tensile or compressive stress regions. The stress distribution is largely uniform across the structure, with localized tensile stress regions appearing at the upper edge of the container walls near the load application point. The slight compression indicated by negative principal stress values occurs on the opposite surfaces due to bending effects under the applied load. This behaviour aligns with expected mechanical response and validates the accuracy of the simulation setup. The refined mesh, consisting of 49,096 nodes and 23,894 elements, ensures precise capture of stress gradients, even in small geometric features. The overall contour plot indicates that the chassis geometry and load path are well-optimized, preventing stress concentration that could lead to fatigue or failure. From a structural performance perspective, the negligible principal stress values confirm that the steel frame is mechanically overdesigned for the given load case, providing ample margin for heavier attachments, increased payload, or operational shocks during field use. This result, together with von Mises and safety factor analyses, provides a complete simulation of the robot’s structural robustness.

**Table 2:** FEA structural analysis results for agricultural robot chassis (Load = 15 N).

Stress Type	Stress Range (MPa)	Interpretation
Von Mises Stress	Max: 0.002Min: 0.000	Indicates overall yield safety for ductile materials. Extremely low stress values confirm large safety margin against yielding.
1 st Principal Stress	Max: 0.002Min: − 0.00059	Represents maximum tensile stress in the structure. Very low values indicate negligible risk of tensile failure.
3rd Principal Stress	Max: ~0.000Min: − 0.002	Represents maximum compressive stress. Low magnitude confirms no risk of buckling or compressive fracture.
Safety Factor	Constant: 15.00	Significantly above standard design requirement (≥ 2), confirming the design is over-engineered for the tested load case.
Mesh Details	49,096 Nodes23,894 Elements	Fine mesh ensures accurate stress resolution in localized areas.

Table 2 summarizes the results of the FEA stress analyses performed on the chassis under a 15 N load. All stress types of exhibit values several orders of magnitude below typical material yield strengths, and the safety factor remains well above design requirements, confirming the structure’s robustness and mechanical safety.

**Fig. 19:**
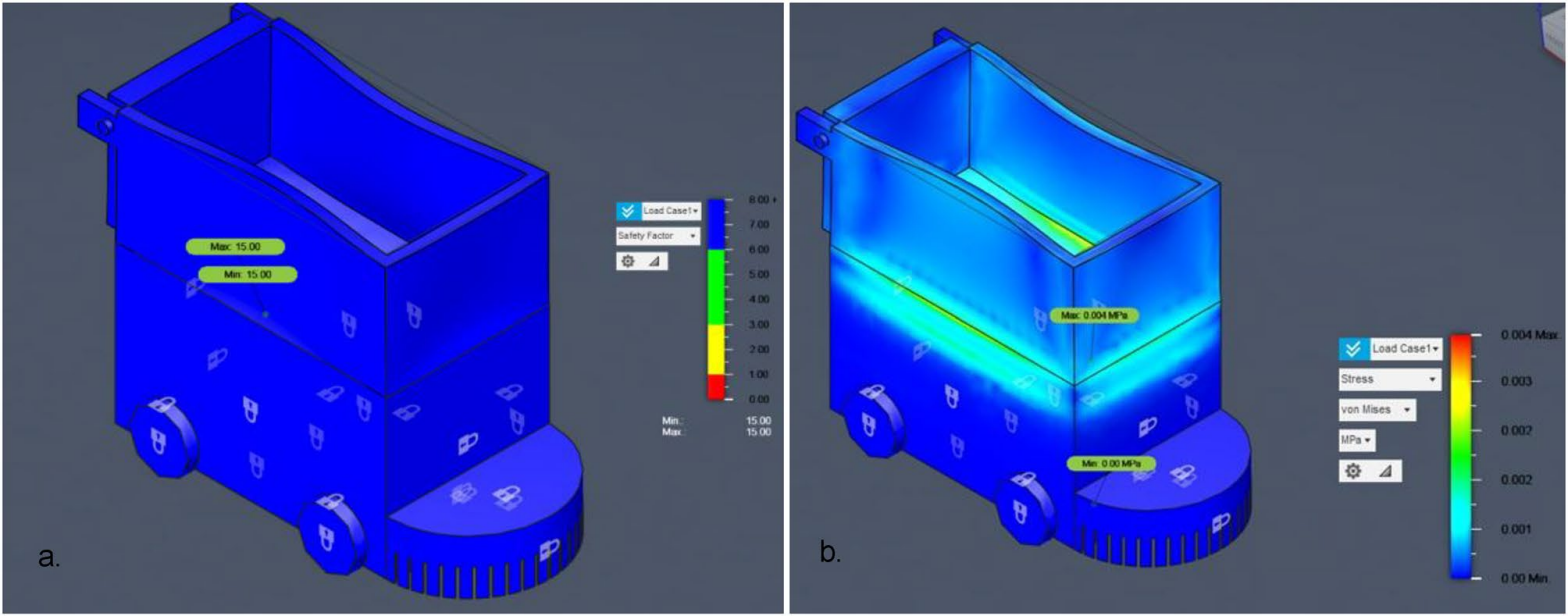
a. Safety Factor for main Bot (Aluminum), and **b**. Stress distribution for main Bot (Aluminum).

The safety factor analysis for a 150 N applied (Fig. 19.a) load indicates a uniform safety factor of 15 across the entire structure. This reflects an extremely conservative and robust design, suggesting that the bot can withstand forces up to 2250 N before failure. No critical stress zones were observed, confirming the structural integrity of the bot under the given loading conditions.

The Von Mises stress analysis shows (Fig. 19.b), maximum stress of only 0.004 MPa under the applied load of 150 N. The stress distribution is well within safe limits, with most regions experiencing minimal stress levels. This indicates that the bot structure is mechanically sound and exhibits no signs of material failure or critical stress concentration under operational loading conditions.

**Fig. 20:**
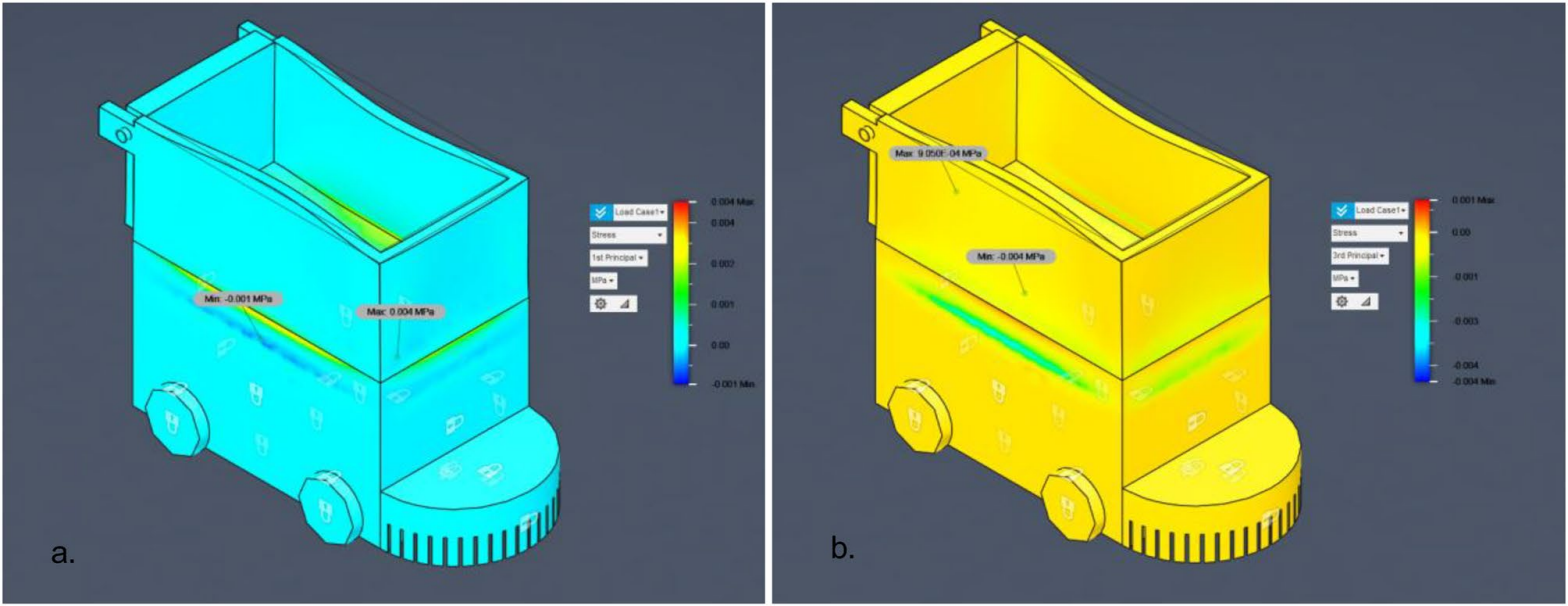
**a**.1st principal stress analysis for main Bot (Aluminum) and, **b**. 3rd principal stress analysis for main Bot (Aluminum).

The 1 st Principal Stress distribution (Fig. 20.a) indicates a maximum tensile stress of 0.004 MPa and a minimum compressive stress of −0.001 MPa under a 150 N load. These values are significantly below the material’s typical yield strength, confirming that the structure experiences minimal stress. The stress pattern is well-distributed with no critical concentrations, affirming the bot’s structural reliability and suitability for the applied loading conditions.

The 3rd Principal Stress results (Fig. 20.b) shows minimum compressive stress of −0.004 MPa and a maximum stress near zero (0.000905 MPa), indicating very low compressive loading in the structure under the 150 N applied force. The distribution suggests that the structure is predominantly under mild compressive stress, with no localized stress concentrations. These values confirm that the design is mechanically sound and well within safe operational limits.

The Finite Element Analysis (FEA) results for the robotic bot under a 150 N load demonstrate excellent structural integrity (Table 3). All evaluated stress parameters, including von Mises, principal stresses, and safety factors fall significantly within acceptable and safe design limits. The uniform stress distribution and consistently high safety factor (15.00) confirm that the bot is mechanically robust and capable of safely withstanding the applied operational forces. Principal stress analysis (1st and 3rd) also confirmed low stress values, with no localized stress concentrations of concern. Based on these results, the estimated failure load capacity of the bot is approximately 2250 N. Thus, no modifications to the structure are necessary from a stress perspective.

**Table 3:** Summary of analysis of Aluminum.

Type	Max Stress (MPa)	Min Stress (MPa)	Critical Observation	Verdict
Von Mises Stress	0.004	0.000	Uniform low stress; well below material yield strength	Safe
1 st Principal Stress	0.004	−0.001	Mild tensile and compressive zones present	Safe
3rd Principal Stress	0.000905	−0.004	Dominated by low compressive stress; no stress spikes	Safe
Safety Factor	15.00	15.00	Uniform high safety factor throughout the structure	Highly safe

### FEA setup and interpretation (Seed-dispenser assembly)

**Fig. 21:**
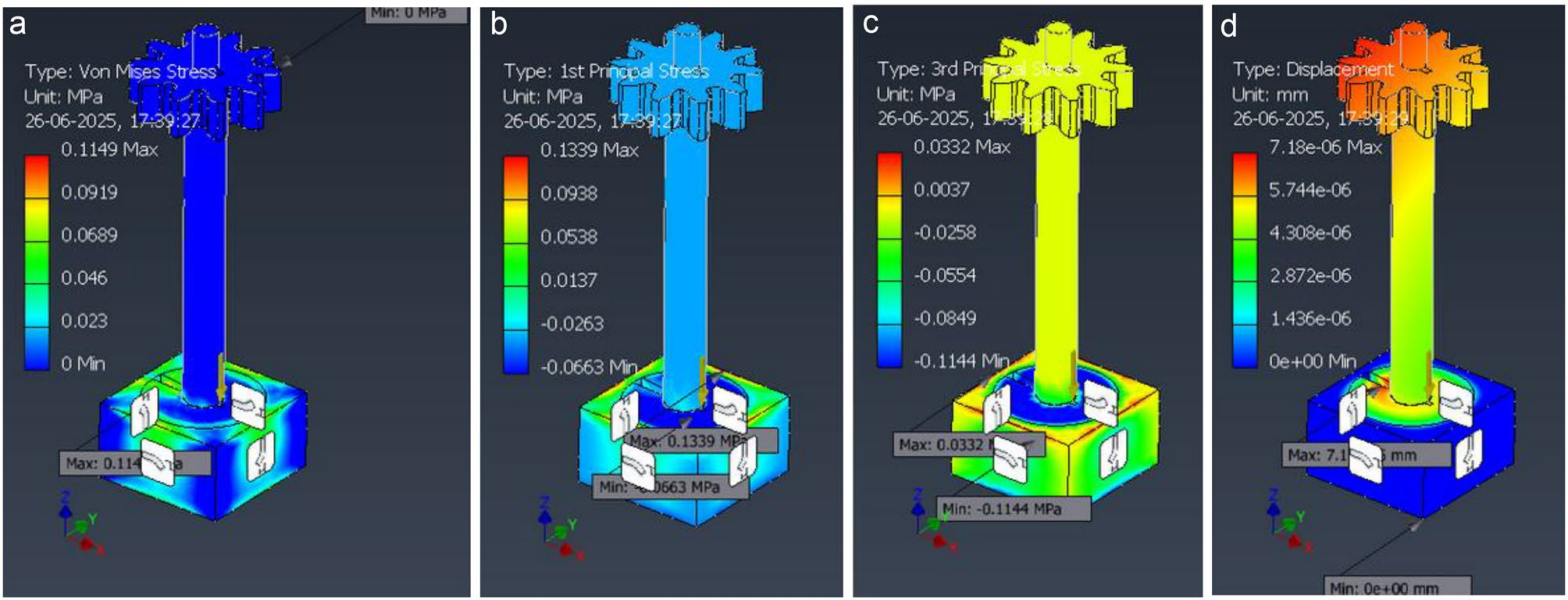
Static structural FEA of the seed-dispenser post/assembly—stress and displacement fields (ANSYS). (**a**) Von Mises stress (MPa), (**b**) 1 st principal (tensile) stress (MPa), (**c**) 3rd principal (compressive) stress (MPa), (**d**) total displacement (mm).

A solid model of the dispenser post, head, and base was analysed using a linear elastic static solver. Unless stated otherwise, material is structural steel with E = 210 GPa, ν = 0.30, and yield strength σy = 250 MPa (aluminium parts use E = 70 GPa, ν = 0.33, σy = 150–250 MPa). The static structural FEA results of the seed-dispenser assembly are presented in Fig. 21, which shows the von Mises stress, principal stress, and displacement fields under representative static loading.

- **Boundary conditions**: The base-plate contact faces to the chassis were constrained with an encastre (fixed) support to provide a conservative upper bound on stresses at the shaft–base fillet. No bolt preload or contact nonlinearity is included in this baseline.

**Fig. 22:**
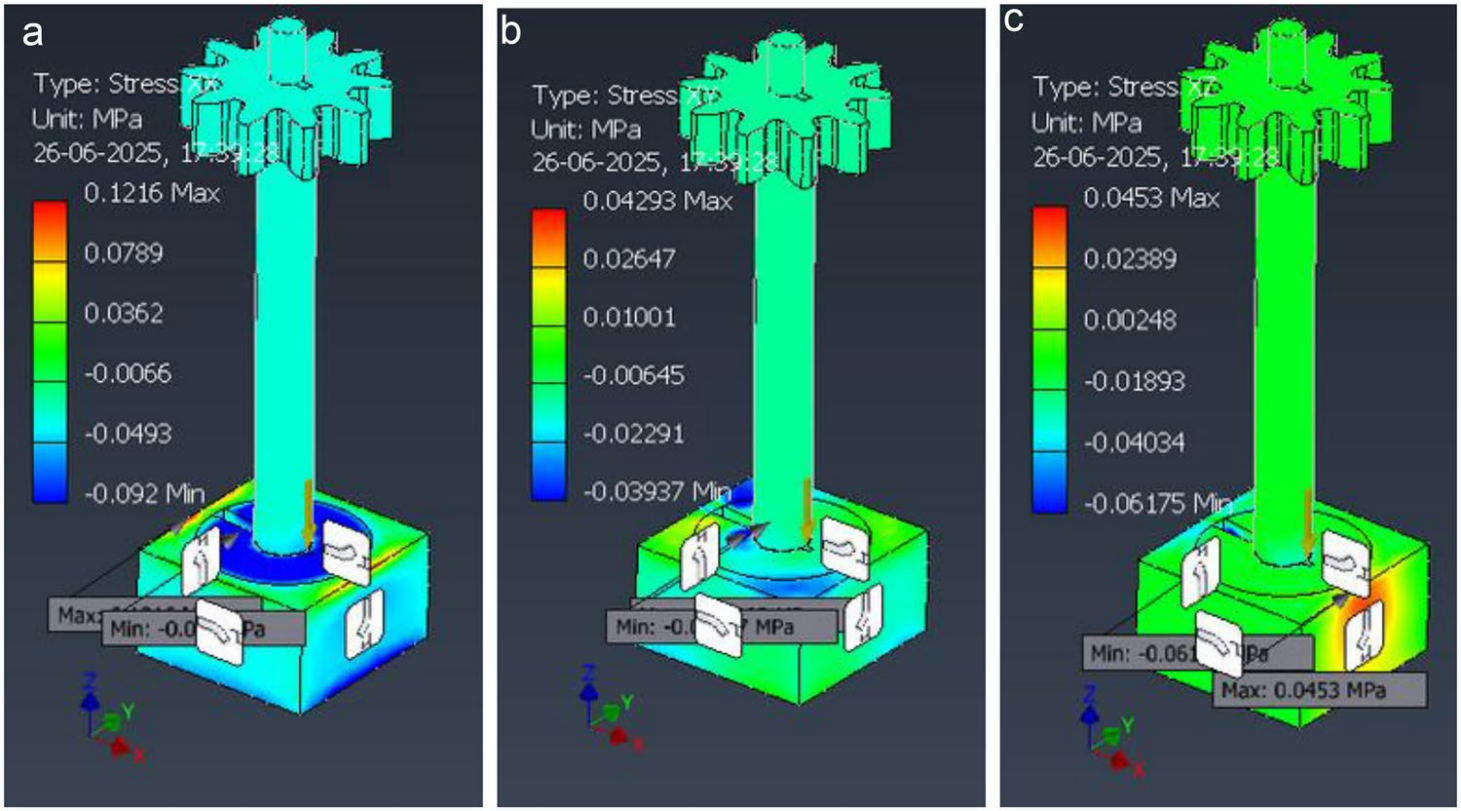
Directional normal stresses in the seed-dispenser assembly under the representative static load. (**a**) σ_x_ (Stress-X), (**b**) σ_y_ (Stress-Y), (**c**) σ_z_ ​ (Stress-Z).

The representative static load case (see FEA Methods) produces low stress levels with local peaks at the shaft–base fillet (σ_{vM, max} ≈ 0.115 MPa, σ_{1,max} ≈ 0.134 MPa, σ_{3,max} ≈ 0.033 MPa). The predicted tip displacement is 7.18 × 10⁻⁶ mm, indicating a very stiff response under the applied static load. Color scales are shown at left of each panel; boundary conditions and load magnitudes correspond to the “representative static loads” described in the text. Figure 22 further illustrates the directional normal stress components (σx, σy, σz) in the seed-dispenser assembly, highlighting localized stress variations around the post–base fillet and confirming bending behavior under applied loads.

**Fig. 23:**
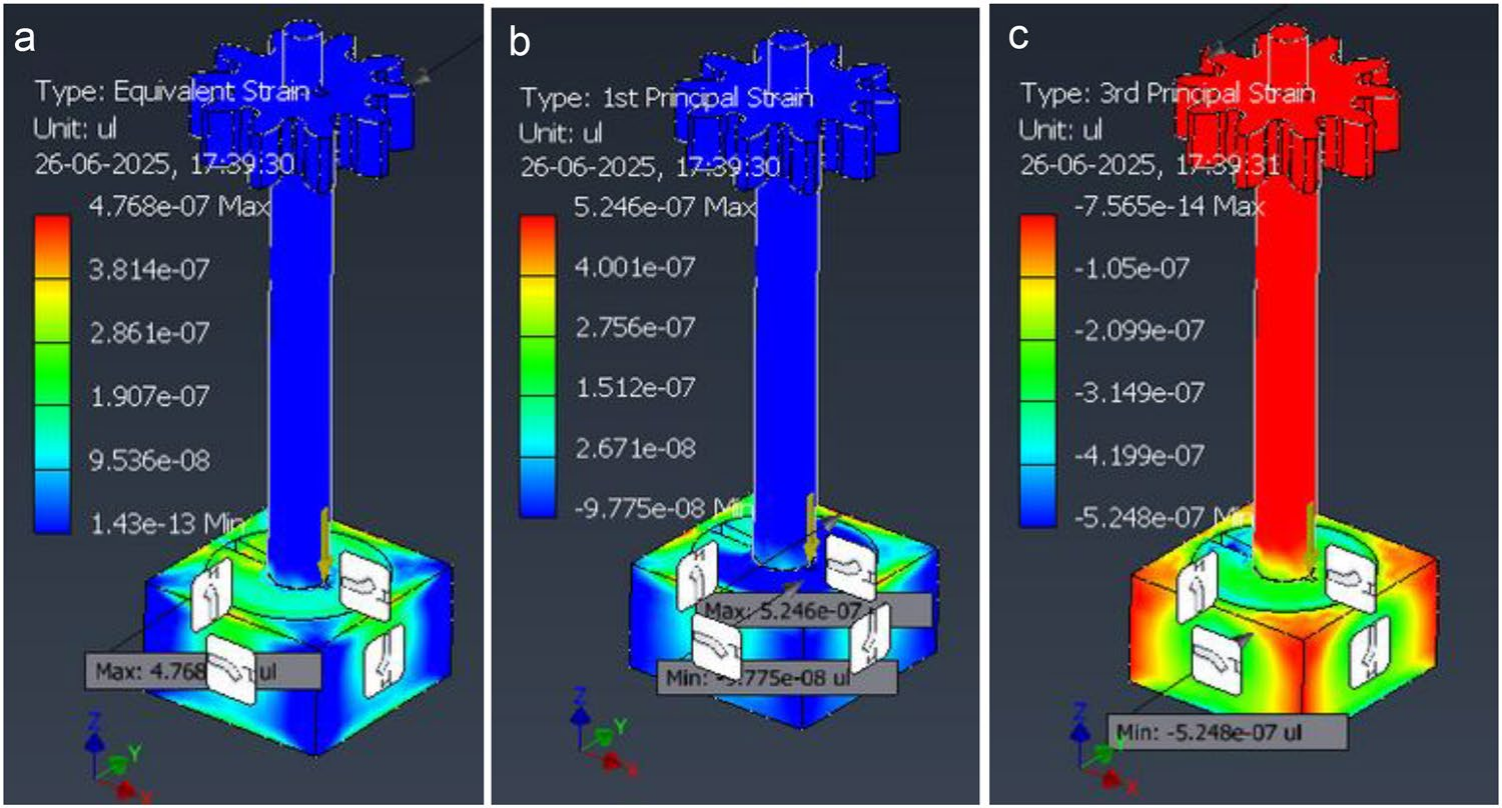
Strain fields in the seed-dispenser assembly under the representative static load. (**a**) Equivalent (von-Mises) strain ε_eqv_​ (unitless; shown as µε scale), (**b**) 1 st principal (tensile) strain ε_1_​, (c) 3rd principal (compressive) strain ε_3_​.

Color scales are in MPa; positive = tension, negative = compression in the global axes (XYZ triad shown). Peak values are small σ_x_,_max_ ≈0.122 MPa, σ_y, max_ ≈ 0.043 MPa, σ_z, max_ ≈ 0.045 MPa with tension/compression lobes around the post–base fillet consistent with lateral bending. These component plots corroborate the von-Mises field (Fig. 22) and help verify bending direction and local bearing in the base plate; load and constraints match the “representative static loads” described in the FEA Methods. The strain distribution within the seed-dispenser assembly is shown in Fig. 23, where the equivalent, principal tensile, and compressive strains remain extremely small, validating the stiffness and elastic response of the design.

Peak values are extremely small ε_eqv, max_ ≈ 4.77 × 10^− 7^(≈ 0.48 µε), ε_1,max_ ≈ 5.25 × 10^− 7^(≈ 0.53 µε), and ε_3,min_ ≈ − 5.25 × 10^− 7^ (≈ − 0.53 µε). Strain localizes at the post–base fillet and along lobe roots, consistent with bending observed in the stress plots (Figs. 24). Using Hooke’s law for steel (E≈210GPa), E _εmax_∼0.10–0.11 MPa, matching the von-Mises peaks and confirming linear-elastic behaviour under these small static loads.

The static structural analysis establishes that the seed-dispenser assembly is mechanically sound and sufficiently stiff for baseline service, before hardware build. It validates the modelling setup (units, materials, boundary conditions), identifies stress-raising features around the post–base fillet and head geometry, and guides small design refinements (fillet radii, wall thickness, fastener locations, bearing supports) without over-building. Critically, it confirms that structural compliance will not disturb metering alignment, so the speed-matched drum maintains spacing accuracy, even when the unit experiences handling or mild lateral loads. The results also define inspection points for maintenance, provide a documented safety margin for reviewer scrutiny and risk assessment, and create a calibrated baseline against which future dynamic cases (impact, snag torque, drop, hitch pull, contact/nonlinear preload) can be added. In short, this FEA de-risks the attachment, informs manufacturable geometry, and supplies defensible evidence that the dispenser will perform as intended when integrated with the robot.

**Table 4:** Comparative analysis of multipurpose agricultural robots.

Robot/System	Key Functionalities/Tasks	Weight/Size	Payload/Tool Capacity	Speed/Coverage	Cost/Notes
MARS (Modular Agricultural Robotic System)^1^	Phenotyping, lightweight sensing, modular tool attachments; precision farming	≈ 500 kg (MARS X)	≥ 150 kg payload	Top speed ~ 1 m/s; good navigation (< 0.05 m error)	Low cost modularity; lighter than tractors
Hefty^2^	Modular reconfigurable robot; manipulation in orchards/vineyards; inspection & pruning	Base Amiga platform	Multiple sensor/manipulator payloads	Precision-oriented; speed modest	Research & field trials; higher cost due to manipulators
SPARROW^7^	Autonomous weed detection & spot spraying; vision-based navigation	Compact differential drive	Sprayer tool & vision payload	Focus on precision spraying, not high speed	Low-cost; targeted for small farms
Low‑Cost Weed Control Robot^14^	Weed detection & spraying; autonomous recharge; narrow-row crops	Compact small footprint	Herbicide spraying payload	Continuous operation with recharge	Prototype built < US$400
Farming GT^15^	Inter-row & in-row mechanical weeding; AI-based crop/weed discrimination	≈ 1.5 ton; 4.7 × 2.5 × 2.0 m	Up to ~ 25 HP equivalent tools	Productivity ~ 10 ha/24 h	Commercial deployment; high cost
Proposed Modular 4WD Robot (This Work)	Grass cutting, leaf crushing, precision seeding; modular toolheads with quick release	≈ 50 kg; 4WD skid-steer	Cutting unit, blower, seeder, crusher modules	Cutting mode 0.15 m²/s; runtime 1.2 h (cutting), 8 h (seeding)	Low-cost fabrication; modularity for rapid task switching

As seen in Table 4, existing systems such as MARS^1^, Hefty^2^, and SPARROW^7^ focus on modular sensing, reconfigurable manipulation, or targeted spraying, but typically lack integrated multi-functionality for cutting, crushing, and seeding. Commercial solutions like Farming GT emphasize high productivity but involve significant weight and cost. In contrast, the proposed 4WD modular robot in this study consolidates multiple ground-maintenance tasks into a lightweight, low-cost, and reconfigurable platform, bridging the gap between research prototypes and practical, deployable systems.

## Conclusion & future work

This work presented the conception, design, and simulation-based evaluation of a modular autonomous robot capable of rapidly switching between grass cutting, leaf crushing, and precision seeding. The platform employs a vertically stacked two-unit architecture that separates the drive/blower subsystem in a steel-framed base from a high-capacity collection chamber, with quick-release mounts supporting interchangeable toolheads. A four-wheel differential-drive (4WD) configuration provides skid-steer agility and stable traversal on uneven ground, while transparent acrylic panels enhance inspection and maintenance. Coverage-path simulations demonstrated that the boustrophedon strategy achieves complete rectangular coverage with minimal redundancy, and differential-drive kinematics support accurate waypoint tracking. Energy-budget modelling predicted practical runtimes of ≈ 1.2 h (cutting), ≈ 2.0 h (crushing), and ≈ 8.0 h (seeding), while robustness simulations confirmed resilience to debris deflection (> 95% rejection), slope climbing (≈ 25° at < 20% slip), and stone-ingestion probability (≈ 10%). Finite-element analysis established very high safety factors (> 15) for both steel and aluminium components under representative static loads. Beyond robotic functionality, composting pathways for collected biomass were outlined to extend the environmental benefits of the platform. Collectively, these results indicate that the modular 4WD robot provides a serviceable, structurally robust, and sustainable solution for campus and small-scale agricultural automation.

Future work will progress toward hardware prototyping, hardware-in-the-loop validation, and field trials to benchmark real cutting throughput, collection efficiency, navigation accuracy, and seeding performance (spacing error, miss/double rates, coefficient of variation). Structural analysis will be extended to dynamic events such as impact, snag torque, drop, hitch pull, and preload contact, providing a more complete mechanical safety assessment. Additional work will address environmental durability (dust, moisture, vibration), refinement of seals and bearings, and weight/efficiency improvements through optimized materials and motor control. On the autonomy side, we plan to integrate onboard perception and learning for selective weeding, obstacle classification, and dynamic re-planning. An operator interface with telemetry, analytics, and remote monitoring will be developed, alongside strategies for multi-robot coordination to scale coverage for larger plots. Finally, valorisation studies of biomass through controlled composting will be experimentally implemented to close the loop between automation, productivity, and sustainability.

## Data Availability

There is no data associated with this research work. This study is a simulation-only design and evaluation. No proprietary experimental datasets were generated. All models are implemented using standard CAD/FEA solvers and Python-based simulation tools. Governing equations, material properties, boundary conditions, mesh sizes, and parameter values are explicitly provided in the manuscript to enable independent reproduction.

## Abbreviations

A: Area cut by the robot m²
v: Forward velocity of the robot m/s
w: Swath width of the cutter head m
h_cut_: Average height of the grass being cut m
ρ_veg_: Bulk density of vegetation (wet grass) kg/m³
m_cut_: Mass of grass cut in one pass kg
Δm_hopper_: Measured increase in hopper mass after cutting kg
η: Collection efficiency – (dimensionless)
(x_i_, y_i_): Robot’s actual position at sample I m
(x_i,ref_, y_i,ref_): Reference position at sample I m
N: Number of position samples used for RMS error calculation
e_rms_: Root-mean-square tracking error m
P_mode_: Power draw in a specific operating mode (cutting/crushing/seeding) W
T_run_: Predicted runtime in a given mode h
µ: Coefficient of friction between wheel and surface – (dimensionless)
s: Slip ratio – (dimensionless)
T: Wheel torque Nm
r: Wheel radius m
θ: Slope inclination angle degrees (°)
α: Debris ingress rate (from Monte Carlo simulation) %
R: Radius of inscribed circle (spiral and concentric paths) m
δ: Effective cutting/swath width m
L_path_: Total path length for coverage m or ft
